# A GBD 2019 study of health and Sustainable Development Goal gains and forecasts to 2030 in Spain

**DOI:** 10.1038/s41598-022-24719-z

**Published:** 2022-12-07

**Authors:** Jeffrey V. Lazarus, Alberto Ortiz, Stefanos Tyrovolas, Esteve Fernández, Danielle Guy, Trenton M. White, Rui Ma, Simon I. Hay, Mohsen Naghavi, Joan B. Soriano, The GBD 2019 Spain Collaborators, Alberto L. García-Basteiro, Jose L. Ayuso-Mateos, Quique Bassat, Fernando G. Benavides, Iago Giné-Vázquez, Josep Maria Haro, Ai Koyanagi, Jose Martinez-Raga, Alicia Padron-Monedero, José L. Peñalvo, Jorge Pérez-Gómez, David Rojas-Rueda, Rodrigo Sarmiento-Suárez, Rafael Tabarés-Seisdedos

**Affiliations:** 1 Barcelona Institute for Global Health (ISGlobal), Hospital Clínic, University of Barcelona, Calle del Rossellón 171, 08036 Barcelona, Spain; 2 Faculty of Medicine and Health Sciences, University of Barcelona, Barcelona, Spain; 3 Department of Medicine, Universidad Autónoma de Madrid, Madrid, Spain; 4 Department of Nephrology and Hypertension, The Institute for Health Research Foundation Jiménez Díaz University Hospital, Madrid, Spain; 5 St. John of God Health Park, San Juan de Dios Sanitary Park, Sant Boi de Llobregat, Spain; 6 Biomedical Research Networking Center for Mental Health Network (CiberSAM), Madrid, Spain; 7 Tobacco Control Unit, Catalan Institute of Oncology- ICO, WHO Collaborating Centre for Tobacco Control, L’Hospitalet de Llobregat, Barcelona, Spain; 8 Tobacco Control Research Group, Bellvitge Biomedical Research Institute -IDIBELL, L’Hospitalet de Llobregat, Barcelona, Spain; 9 Department of Clinical Sciences, School of Medicine and Health Sciences, Campus of Bellvitge, University of Barcelona, Barcelona, Spain; 10 Center for Biomedical Research in Respiratory Diseases Network (CIBERES), Instituto de Salud Carlos III, Madrid, Spain; 11 Institute for Health Metrics and Evaluation, University of Washington, Seattle, WA USA; 12 Department of Health Metrics Sciences, School of Medicine, University of Washington, Seattle, WA USA; 13 Hospital Universitario de la Princesa, Universidad Autónoma de Madrid, Madrid, Spain; 14 Centro de Investigação em Saúde de Manhiça (CISM), Maputo, Mozambique; 15 Centro de Investigación Biomédica en Red de Enfermedades Infecciosas (CIBERINFEC), Barcelona, Spain; 16 Department of Psychiatry, Universidad Autónoma de Madrid, Madrid, Spain; 17 Biomedical Research Networking Center for Mental Health Network (CiberSAM), Instituto de Salud Carlos III (Carlos III Health Institute), Madrid, Spain; 18 Catalan Institution for Research and Advanced Studies (ICREA), Barcelona, Spain; 19 Department of Experimental and Health Sciences, Pompeu Fabra University, Barcelona, Spain; 20 Center for Research in Occupational Health, Pompeu Fabra University, Barcelona, Spain; 21 Research, Innovation and Teaching Unit, San Juan de Dios Sanitary Park, Sant Boi de Llobregat, Spain; 22 Research Unit, Universitat de Barcelona (University of Barcelona), Barcelona, Spain; 23 Psychiatry Department, Hospital Universitario Doctor Peset, Valencia, Spain; 24 Department of Medicine, University of Valencia, Valencia, Spain; 25 National School of Public Health, Instituto de Salud Carlos III (Carlos III Health Institute), Madrid, Spain; 26 Department of Public Health, Institute of Tropical Medicine, Antwerp, Belgium; 27 Friedman School of Nutrition Science and Policy, Tufts University, Boston, MA USA; 28 Departamento de Didáctica de la Expresión Musical, Plástica y Corporal (Department of Didactics of Musical, Plastic, and Corporal Expression), University of Extremadura, Cáceres, Spain; 29 Department of Environmental and Radiological Health Sciences, Colorado State University, Fort Collins, CO USA; 30 Department of Health and Society, University of Applied and Environmental Sciences, Bogotá, Colombia

**Keywords:** Diseases, Risk factors

## Abstract

This study aimed to report mortality, risk factors, and burden of diseases in Spain. The Global Burden of Disease, Injuries, and Risk Factors 2019 estimates the burden due to 369 diseases, injuries, and impairments and 87 risk factors and risk factor combinations. Here, we detail the updated Spain 1990–2019 burden of disease estimates and project certain metrics up to 2030. In 2019, leading causes of death were ischaemic heart disease, stroke, chronic obstructive pulmonary disease, Alzheimer’s disease, and lung cancer. Main causes of disability adjusted life years (DALYs) were ischaemic heart disease, diabetes, lung cancer, low back pain, and stroke. Leading DALYs risk factors included smoking, high body mass index, and high fasting plasma glucose. Spain scored 74/100 among all health-related Sustainable Development Goals (SDGs) indicators, ranking 20 of 195 countries and territories. We forecasted that by 2030, Spain would outpace Japan, the United States, and the European Union. Behavioural risk factors, such as smoking and poor diet, and environmental factors added a significant burden to the Spanish population’s health in 2019. Monitoring these trends, particularly in light of COVID-19, is essential to prioritise interventions that will reduce the future burden of disease to meet population health and SDG commitments.

## Introduction

Spain’s public health system is primarily funded by public sources and covers over 99% of the population, with primary care serving as the first point of access for nearly all patients^1^. Health system management has been decentralised since 2002, with devolved authority at the regional (*comunidad autónoma*) level. The national government is responsible for the overall coordination and monitoring of health system performance and for contributing to health equity among regions through, for instance, its monitoring efforts and funding^1^. In addition to the functioning of the national health system, which plays a crucial role in determining and maintaining population health^2^, health in Spain is shaped by several social determinants including income, educational attainment level, household structure, and gender^3–5^.

To improve health system monitoring and public health research efforts to reduce health inequalities, we assess the state of health in Spain using data from the 2019 Global Burden of Diseases, Injuries, and Risk Factors (GBD) Study, which measures communicable, maternal, neonatal, and nutritional diseases (CMNNDs), non-communicable diseases (NCDs), and injuries among populations around the world, in a comparable format. The GBD facilitates the monitoring and comparison of health indicators within and among countries to help achieve national^1^ and global^6^ health targets, including the health-related United Nations’ Sustainable Development Goals (SDGs)^7^.

Previous Spain-specific GBD studies were published in 2014^8^ and 2018^9^, in addition to 11 reports employing GBD methodology and/or data in Spain (see Supplementary Appendix 1). Here, using GBD 2019 data, we assess the state of health in Spain immediately before the COVID-19 pandemic and present the results of trends from 1990 to 2019. In addition, we develop projections for meeting the SDG targets by 2030, in order to better identify unmet health needs, inform appropriate interventions, and provide relevant insight into future health trends. This manuscript was produced as part of the GBD Collaborator Network and in accordance with the GBD Protocol.

## Methods

### Overview

The GBD 2019 estimated disease burden worldwide and in Spain from 369 diseases, injuries, and impairments, as well as 87 risk factors and combinations of risk factors, through systematic assessment of published, publicly available, and nationally-contributed data on incidence, prevalence, and mortality, for a mutually exclusive and collectively exhaustive list of diseases and injuries^10,11^. The GBD 2019 produced age- and sex-specific estimates globally, regionally, and for 204 countries and territories (including selected subnational units) using the comparative risk assessment (CRA) framework of cause-specific risk factor exposure, morbidity, and mortality attributable to these risks, and a range of health system characteristics, with details of this methodology being available elsewhere^11^. The CRA framework systematically evaluates changes in population health that would arise from modifying the population distribution of exposure to a single risk factor or groups of risk factors. For this study, summary measures were computed using standardised and validated approaches that adjust for major sources of bias (see Supplementary Appendix 2). Notably, GBD 2019 used newly available risk factors for non-optimal global earth temperatures, measuring the environmental effects of changes in ambient temperatures on disease outcomes, and standardisation methods to improve the quality of available statistical data to calculate these risks^12^. Data on life expectancy, years of life lost (YLLs), years lived with disability (YLDs), and disability-adjusted life-years (DALYs) in Spain from 1990 to 2019 were extracted from GBD 2019, full details of which are published elsewhere^10^. Inequality in disease burden was examined using the Gini coefficient.

The GBD estimates levels and trends in exposure, attributable deaths, and attributable DALYs, using the CRA framework. These variables are disaggregated by age group, sex, year, and level 1 category of risks (i.e. behavioural, environmental and occupational, and metabolic) or clusters of risks from 1990 to 2019^12^. The GBD also uses various statistical models to address data quality issues, to establish the disease burden and attributable risk for each disease, injury, and impairment. The GBD classifies diseases and injuries in a hierarchy containing four levels, each with increasing details of risk. This study reports those at level three, which represent specific causes of disease and injury (e.g. tuberculosis and road injuries). For example, ischemic stroke is a level 4 cause in the level 3 stroke group, which is in the level 2 cardiovascular diseases group. Level three was chosen for this analysis because level two is considered as ‘too aggregate’ to suitably capture certain diseases, while level four is ‘too detailed’ and is primarily used for specific disease papers. For risk factors, the GBD 2019 estimation of attributable burden followed the general framework established for CRA used in GBD since 2002, with changes for 2019 detailed elsewhere^11^. This study presents life expectancy, main causes of death, YLLs, YLDs, and DALYs, along with their causes, stratified by sex at the national level in Spain. As stated above, non-optimal earth temperature is a new risk factor from GBD 2019; it represents an aggregate of the burden attributable to low and high temperatures. Heat and cold effects relate to effects above and below the theoretical minimum risk exposure level (TMREL). The population-weighted mean TMREL is 25.6 °C^13,14^.

### Data obtention and processing

The GBD estimation process is based on identifying multiple relevant data sources for each disease or injury. The exact data sources for Spanish estimates are accessible at its Institute of Health Metrics and Evaluation (IHME) country profile^15^. The primary sources for the cause of death data were the Mortality Information System hosted by the *Instituto Nacional de Estadística (INE)* and the World Health Organization (WHO)^16^. IHME collects data to calculate relative risks from cohort studies, randomised control trials, literature reviews, and other sources (see Supplementary Appendix 2). The GBD uses this data and corrects for the underreporting of deaths and garbage codes (i.e. anything marked as a cause of death that cannot be an underlying cause or is an unspecified cause)^17^ based on the medical literature, expert opinions, and statistical techniques used to assign the most probable causes of death to each item^18^.

### Sustainable Development Goal indicators

We also report IHME estimates for Spain’s health-related SDG index score. This measure is the overall measure of all health-related SDGs. The SDG assessment measured progress on 41 health-related SDG indicators, including smoking prevalence, air pollution, intimate partner violence, and vaccine coverage, from 1990 to 2017 for 195 countries and territories^7^. To construct the health-related SDG index, the value for each indicator was transformed on a scale from 0 to 100. This was based on 1000 observed or projected random samples calculated from 1990 to 2030, to reduce sensitivity to extreme outliers in the overall sample. For this scale, 0 represents the 2.5th percentile and 100 represents the 97.5th percentile. The geometric mean of the scaled indicators was also taken for each target.

To generate projections through 2030, IHME used a forecasting framework that compiles the impacts of independent drivers of population health into the future, to assess the probability of each country’s attainment for defined SDG targets. As a tool for projections, we used a meta-regression Bayesian, regularised, trimmed (MR-BRT) mixed effects model, that provides an easy interface for formulating and solving common linear and non-linear mixed effects models used in the most recent GBD iterations^7^. This framework drew estimates from the broader GBD study and weighted averages of indicator-specific and country-specific annualised rates of change from 1990 to 2017^7^. The impact of the COVID-19 pandemic is not part of this analysis, with such projections having been estimated elsewhere^19^.

We derived 95 percent uncertainty intervals of all estimates using simulation methods, which resemble but are not the same as 95% confidence intervals. We constructed 1000 draws with the required correlation structure between variables separately for each cause, and the 2.5th percentile and the 97.5th percentile of expected events were taken to be the lower and upper bounds of the corresponding uncertainty interval. These ranges provide guidance on uncertainty in the underlying cause-specific rates, as expressed in terms of expected events in the population^7^. All methods were performed in accordance with the relevant guidelines and regulations; this study conforms to international ethical standards, including the 1975 Declaration of Helsinki.

## Results

**Figure 1:**
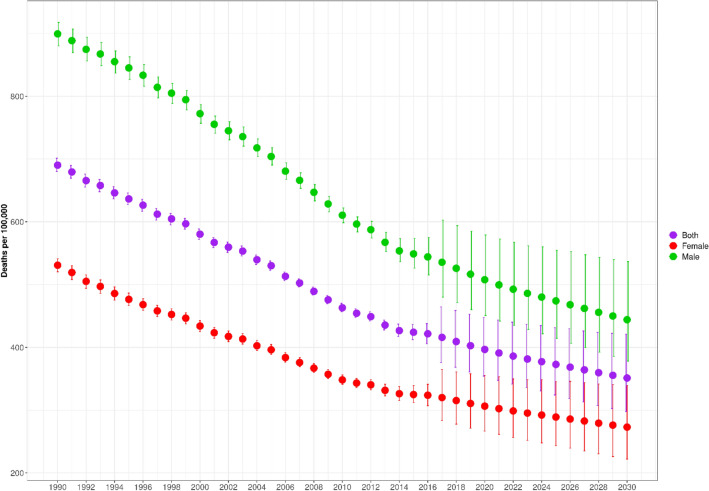
Death rate (per 100,000) (age-standardised) from 1990 and projected changes to 2030, in both sexes.

In 2019, Spain had a total population of 46.0 million people (51.1% female). Spain has an ageing population (see Supp Fig. S1), which is expected to continue through 2030. By 2030, life expectancy in Spain is projected to reach 84.8 years (uncertainty interval (UI): 83.1–86.0); 87.2 (UI: 85.3–88.6) for females and 82.3 (UI: 80.6–83.7) for males (see Supp Fig. S2). Recent projections show that, in the absence of health system and social impacts from COVID-19, Spain would have continued to experience declines in death rates for both sexes, with a more rapid decline for males (Fig. 1).

### Mortality and morbidity

The main causes of death and YLLs by sex are shown in Table 1. In 2019, an estimated 428,577 deaths (UI: 421,705–435,908) occurred in Spain. NCDs caused 92.0% (UI: 91.3–93.0) of all deaths. Of the NCDs, the highest-ranking specific causes of death were ischaemic heart disease (IHD) (53,632, UI: 46,434–59,832), stroke (37,092, UI: 30,981–42,048), chronic obstructive pulmonary disease (COPD) (31,245, UI: 25,155–36,629), Alzheimer’s disease and other dementias (29,208, UI: 7,447–72,045), and tracheal, bronchus, and lung cancer (24,523, UI: 22,753–25,958). Of the cancers, tracheal, bronchus, and lung cancer caused the most frequent deaths with 24,523 (UI: 22,753–25,958), followed by colorectal cancers with 20,011 (UI: 17,768–21,746). Breast cancer accounted for 7981 deaths (UI: 7002–8763) in females and prostate cancer accounted for 8406 deaths (UI: 6964–12,143) in males.

**Table 1:** Causes of death and years of life lost (YLLs) in all ages, by sex, in Spain in 2019.

Cause of death or injurys	Deaths									YLL								
	Both sexes	Both sexes, lower UI	Both sexes, upper UI	Males	Males, lower UI	Males, upper UI	Females	Females, lower UI	Females, upper UI	Both sexes	Both sexes, lower UI	Both sexes, upper UI	Males	Males, lower UI	Males, upper UI	Females	Females, lower UI	Females, upper UI
All causes	428,577	421,705	435,908	213,851	210,262	217,675	214,726	211,443	218,233	6,330,140	6,205,667	6,464,235	3,644,301	3,569,781	3,724,607	2,685,839	2,635,886	2,739,737
**Communicable, maternal, neonatal, and nutritional diseases**	18,134	15,251	20,376	8682	7660	9601	9452	7528	10,868	270,190	241,863	293,260	151,467	138,253	163,006	118,723	102,318	131,322
Tuberculosis	379	330	425	219	195	243	160	132	186	6535	5917	7158	4166	3807	4525	2369	2024	2688
HIV/AIDS	679	640	718	544	507	581	135	125	145	28,252	26,647	29,847	22,223	20,683	23,743	6029	5617	6499
Diarrheal diseases	1213	953	1485	421	343	511	792	598	988	13,167	10,814	15,595	5329	4497	6194	7838	6181	9582
Lower respiratory infections	14,184	11,739	16,134	6671	5783	7456	7513	5871	8720	155,930	135,227	173,139	83,183	74,310	91,573	72,747	82,501	59,648
Other infectious diseases	647	562	796	346	286	446	301	253	404	16,454	14,528	18,748	9317	7764	11,260	7137	6110	8290
Neglected tropical diseases and malaria	17	8	57	10	4	37	7	3	20	508	165	2917	330	88	2001	178	53	912
Maternal disorders	15	13	16	0	0	0	15	13	16	778	704	857	0	0	0	778	704	857
Neonatal disorders	483	387	591	274	216	337	209	165	252	42,882	34,379	52,457	24,316	19,133	29,941	18,566	14,669	22,382
Nutritional deficiencies	467	377	542	179	150	206	288	221	351	4804	4118	5404	2222	1961	2488	2582	2076	3064
Other direct maternal disorders	9	8	10	0	0	0	9	8	10	480	416	546	0	0	0	480	416	546
Other neonatal disorders	149	115	191	84	64	110	65	49	83	13,230	10,189	16,921	7484	5660	9786	5746	4351	7375
Other nutritional deficiencies	165	103	217	62	31	85	103	52	140	1725	1125	2146	804	405	1044	921	480	1245
**Non-communicable diseases**	394,268	387,248	401,816	195,228	191,686	198,867	199,040	195,481	203,204	5,643,796	5,530,194	5,762,752	3,193,588	3,126,582	3,262,996	2,450,208	2,403,065	2,502,739
Neoplasms	125,834	114,911	132,579	75,562	70,751	79,398	50,272	43,763	53,962	2,330,341	2,191,157	2,425,106	1,431,450	1,365,586	1,489,026	898,891	826,133	947,034
Esophageal cancer	2331	2105	2571	1927	1749	2122	404	342	464	50,141	45,504	55,135	43,307	39,380	47,835	6834	5981	7685
Stomach cancer	7240	6461	7873	4138	3814	4471	3102	2604	3467	125,169	115,519	135,123	77,457	71,676	83,082	47,712	42,389	52,020
Liver cancer	4973	4472	5437	3305	2956	3677	1668	1401	1888	96,343	86,958	105,862	69,769	61,928	78,264	26,574	23,225	29,682
Larynx cancer	1501	1368	1651	1411	1282	1556	90	68	105	32,135	29,240	35,561	30,202	27,325	33,504	1933	1439	2247
Tracheal, bronchus, and lung cancer	24,523	22,753	25,958	19,466	17,978	20,712	5057	4479	5583	516,790	484,690	545,363	404,606	376,778	429,621	112,184	100,357	123,584
Breast cancer	8075	7100	8851	94	77	114	7981	7002	8763	163,797	150,241	175,267	1822	1526	2148	161,975	148,544	173,454
Cervical cancer	1149	806	1286	0	0	0	1149	806	1286	26,177	17,846	29,075	0	0	0	26,177	17,846	29,075
Uterine cancer	1609	1395	1798	0	0	0	1609	1395	1798	27,520	24,281	30,592	0	0	0	27,520	24,281	30,592
Prostate cancer	8406	6964	12,143	8406	6964	12,143	0	0	0	107,939	91,050	159,849	107,939	91,050	159,849	0	0	0
Colon and rectum cancer	20,011	17,768	21,746	11,297	10,210	12,310	8714	7295	9796	325,536	298,506	347,973	194,944	178,507	209,989	130,592	115,308	142,740
Lip and oral cavity cancer	1578	1428	1705	1062	959	1156	516	443	582	32,948	30,099	35,512	24,209	21,796	26,641	8739	7737	9639
Nasopharynx cancer	275	243	306	204	180	229	71	61	80	7058	6241	7895	5464	4798	6167	1594	1405	1785
Other pharynx cancer	851	751	957	752	664	844	99	86	114	22,154	19,560	25,026	19,734	17,385	22,347	2420	2095	2785
Gallbladder and biliary tract cancer	1567	1184	1771	648	396	749	919	680	1080	24,712	18,954	27,478	11,141	7006	12,733	13,571	10,382	16,172
Pancreatic cancer	7900	6998	8688	3870	3504	4221	4030	3387	4541	147,446	134,177	161,020	80,297	73,126	87,186	67,149	59,093	74,490
Malignant skin melanoma	1105	575	1285	599	264	721	506	227	601	24,185	12,877	27,847	13,567	6101	16,292	10,618	4722	12,463
Non-melanoma skin cancer	817	696	916	460	403	521	357	283	420	9939	8782	11,058	6395	5730	7227	3544	2932	4051
Ovarian cancer	2378	1978	2675	0	0	0	2378	1978	2675	49,909	43,012	55,636	0	0	0	49,909	43,012	55,636
Testicular cancer	59	51	68	59	51	68	0	0	0	1977	1718	2267	1977	1718	2267	0	0	0
Kidney cancer	2957	2601	3297	1972	1769	2178	985	822	1131	56,092	50,612	61,515	39,292	35,745	43,055	16,800	14,648	18,712
Bladder cancer	6580	5750	7400	5282	4713	5913	1298	1054	1494	95,411	85,987	106,029	79,123	71,660	88,128	16,288	13,880	18,285
Brain and central nervous system cancer	3083	1637	3679	1783	927	2082	1300	560	1676	81,076	44,415	94,313	49,079	26,708	56,649	31,997	14,510	40,016
Thyroid cancer	418	356	465	177	118	203	241	203	276	7581	6481	8339	3608	2386	4135	3973	3451	4455
Mesothelioma	481	433	541	364	328	402	117	92	163	9481	8589	10,514	7131	6439	7893	2350	1747	3110
Hodgkin lymphoma	241	186	283	136	96	168	105	68	130	6226	4952	7255	3718	2686	4578	2508	1739	3202
Non-Hodgkin lymphoma	3275	2829	3703	1696	1443	1992	1579	1289	1864	60,938	54,043	68,143	34,680	29,579	40,775	26,258	22,346	30,493
Multiple myeloma	2372	1913	2635	1204	878	1377	1168	914	1354	38,998	33,558	43,304	20,781	16,175	23,634	18,217	15,029	20,858
Leukemia	4190	3707	4629	2326	2073	2590	1864	1512	2147	78,647	72,082	84,430	45,337	41,568	49,669	33,310	29,028	36,679
Other neoplasms	2129	1647	2940	1121	722	1856	1008	798	1181	26,594	20,620	38,145	15,103	9993	25,955	11,491	9521	13,071
Other malignant neoplasms	3756	3121	4152	1800	1426	2003	1956	1635	2211	77,423	65,751	84,982	40,768	32,690	45,193	36,655	31,891	40,714
Cardiovascular diseases	131,492	112,385	142,226	56,873	51,908	60,348	74,619	60,496	83,181	1,604,962	1,428,611	1,712,277	852,243	800,520	895,495	752,719	632,892	825,500
Rheumatic heart disease	2492	1988	3056	678	561	811	1814	1406	2245	32,296	26,296	38,692	10,359	8674	12,235	21,937	17,607	26,617
Ischemic heart disease	53,632	46,434	59,832	26,869	24,591	28,963	26,763	21,674	31,198	702,210	638,688	765,172	431,269	405,669	459,990	270,941	228,072	309,641
Stroke	37,092	30,981	42,048	14,715	12,925	16,311	22,377	17,828	26,330	426,742	371,628	473,043	199,661	180,572	218,634	227,081	187,375	261,456
Hypertensive heart disease	8727	4525	10,420	2240	1102	2632	6487	3178	7859	82,066	46,619	96,282	25,868	14,372	29,825	56,198	29,170	67,328
Cardiomyopathy and myocarditis	6442	5210	7436	3080	2215	3664	3362	2535	4114	88,586	70,708	100,860	53,824	37,944	63,155	34,762	27,756	41,840
Atrial fibrillation and flutter	7378	5887	9278	2398	1610	3277	4980	3863	6648	71,702	58,337	90,498	26,277	17,612	36,229	45,425	35,982	59,355
Aortic aneurysm	2401	2107	2684	1820	1602	2045	581	479	672	39,718	35,416	43,869	31,398	28,159	34,915	8320	7194	9401
Peripheral artery disease	1890	885	3505	989	339	2286	901	292	1947	21,008	9729	40,461	12,656	4335	29,368	8352	2678	18,300
Endocarditis	1781	872	2273	597	229	782	1184	579	1536	23,413	11,143	29,209	9904	3878	12,665	13,509	6605	16,980
Other cardiovascular and circulatory diseases	3478	2917	3882	1256	1114	1391	2222	1755	2541	47,870	42,007	52,263	21,455	19,411	23,505	26,415	21,879	29,930
Chronic respiratory diseases	36,560	29,180	42,491	21,824	18,455	24,963	14,736	9076	18,872	421,093	360,359	475,441	270,935	235,492	305,425	150,158	104,121	185,037
Chronic obstructive pulmonary disease	31,246	25,155	36,629	19,593	16,386	22,484	11,653	7326	15,404	348,086	294,884	395,506	236,616	204,628	267,997	111,470	77,522	141,957
Pneumoconiosis	299	243	363	274	221	335	25	15	36	3934	3285	4686	3635	3027	4362	299	199	412
Asthma	1122	798	1444	183	149	221	939	623	1242	13,857	10,735	16,871	2850	2394	3375	11,007	8141	13,821
Interstitial lung disease and pulmonary sarcoidosis	3598	1732	5236	1641	746	2272	1957	789	3095	50,202	27,556	67,424	25,284	12,359	33,678	24,918	12,476	35,731
Other chronic respiratory diseases	296	219	510	134	93	334	162	99	300	5014	3633	9802	2550	1765	7196	2464	1670	5023
Cirrhosis and other chronic liver diseases	8218	7418	9134	5234	4787	5787	2984	2471	3513	177,289	163,281	191,551	127,155	116,900	138,367	50,134	43,937	56,117
Digestive diseases (except cirrhosis)	23,212	20,269	25,329	11,618	10,724	12,489	11,594	9487	13,048	359,216	329,378	383,485	217,733	204,340	231,359	141,483	121,211	155,357
Appendicitis	127	98	181	67	45	109	60	44	79	1919	1467	2725	1122	711	1826	797	591	1028
Paralytic ileus and intestinal obstruction	2940	2127	3509	1289	902	1552	1651	1096	2049	33,762	25,912	39,125	16,752	12,344	20,005	17,010	12,028	20,556
Inguinal, femoral, and abdominal hernia	753	621	908	320	270	381	433	331	544	8488	7247	9960	3949	3404	4571	4539	3573	5584
Inflammatory bowel disease	377	303	600	184	145	298	193	148	355	5929	5035	8033	3260	2680	4551	2669	2184	4214
Vascular intestinal disorders	3650	3047	4370	1438	1236	1696	2212	1757	2718	42,257	36,313	49,179	19,331	16,966	22,201	22,926	18,772	27,267
Gallbladder and biliary diseases	2916	1954	3693	1177	625	1501	1739	1159	2225	31,115	21,149	38,774	14,282	7720	17,877	16,833	11,670	21,142
Pancreatitis	1592	1359	1820	787	679	924	805	619	971	25,334	22,230	29,154	15,056	13,058	18,279	10,278	8467	12,205
Other digestive diseases	1760	1183	2316	689	420	948	1071	672	1538	21,460	14,090	28,857	10,049	6094	14,196	11,411	7179	16,088
Neurological disorders	38,552	17,408	80,595	13,570	7337	26,950	24,982	9863	54,358	414,317	216,717	817,992	168,841	104,728	308,364	245,476	112,632	510,352
Alzheimer’s disease and other dementias	29,208	7447	72,045	8297	2005	22,012	20,911	5457	50,985	268,318	68,024	681,226	84,376	20,281	228,500	183,942	47,782	455,487
Parkinson's disease	6137	5325	6641	3654	3260	3971	2483	2018	2767	71,320	63,628	76,769	43,967	39,991	47,607	27,353	22,926	30,166
Idiopathic epilepsy	634	464	709	310	279	341	324	160	387	14,563	12,149	15,797	8213	7489	8962	6350	4069	7164
Multiple sclerosis	270	218	450	104	80	192	166	122	290	7715	6296	12,671	3025	2314	5611	4690	3497	8063
Motor neuron disease	1118	989	1244	595	521	668	523	445	597	25,246	22,448	27,999	14,023	12,381	15,670	11,223	9670	12,829
Other neurological disorders	1185	1054	1310	610	551	674	575	498	650	27,153	24,894	29,426	15,235	13,939	16,818	11,918	10,685	13,046
Mental disorders	3	2	4	0	0	0	3	2	4	170	126	222	2	1	2	168	124	220
Eating disorders	3	2	4	0	0	0	3	2	4	170	126	222	2	1	2	168	124	220
Substance use disorders	1157	1050	1277	886	796	993	271	242	299	41,699	37,380	46,799	33,844	30,089	38,486	7855	7099	8762
Alcohol use disorders	429	385	473	359	317	400	70	61	79	12,789	11,504	14,220	10,627	9387	11,984	2162	1860	2484
Drug use disorders	728	641	838	527	453	624	201	176	230	28,910	24,974	33,803	23,217	19,768	27,755	5693	5027	6624
Diabetes and kidney diseases	24,786	20,908	27,367	10,211	9174	11,063	14,575	11,669	16,578	271,861	238,541	294,551	128,244	118,509	137,614	143,617	118,921	159,732
Diabetes mellitus	10,136	8571	11,228	4094	3669	4500	6042	4754	6972	119,823	104,885	130,388	57,415	52,423	62,555	62,408	51,516	70,563
Acute glomerulonephritis	6	4	7	3	2	4	3	2	4	66	52	82	36	27	49	30	21	40
Chronic kidney disease	14,645	12,084	16,737	6114	5367	6837	8531	6673	9967	151,972	131,366	170,093	70,793	63,921	77,854	81,179	66,503	93,252
Urinary diseases and male infertility	5837	3546	6814	2167	1156	2635	3670	2098	4452	59,486	39,047	68,236	24,190	13,541	28,762	35,296	21,149	41,719
Gynecological diseases	32	24	39	0	0	0	32	24	39	532	428	646	0	0	0	532	428	646
Hemoglobinopathies and hemolytic anemias	413	345	488	164	146	181	249	191	319	6392	5556	7384	2788	2517	3049	3604	2924	4503
Endocrine, metabolic, blood, and immune disorders	2690	1686	3091	1087	651	1281	1603	778	1909	51,241	36,107	58,306	25,849	16,359	32,119	25,392	13,310	29,216
Upper digestive system diseases	879	715	1078	432	355	525	447	345	560	11,664	9848	13,605	6778	5631	8121	4886	3996	5860
Musculoskeletal disorders	1375	1019	2157	378	294	567	997	666	1770	19,097	14,914	31,912	5664	4634	9574	13,433	9318	24,597
Rheumatoid arthritis	347	260	583	98	77	161	249	165	466	5573	4300	9796	1729	1352	3063	3844	2722	7423
Other musculoskeletal disorders	1028	748	1600	280	210	405	748	493	1315	13,524	10,384	22,147	3935	3184	6466	9589	6719	17,180
Other non-communicable diseases	9637	6524	10,894	3783	2509	4354	5854	3667	6778	163,176	131,647	180,412	77,915	61,284	89,201	85,261	64,157	95,824
Congenital birth defects	628	546	779	342	275	445	286	235	377	42,364	36,514	54,445	23,120	18,681	30,914	19,244	16,004	26,050
Decubitus ulcer	679	252	891	186	29	298	493	161	664	6269	2558	8429	1980	309	3440	4289	1459	5672
Other skin and subcutaneous diseases	46	24	80	17	6	33	29	12	57	581	315	988	234	89	468	347	160	682
Sudden infant death syndrome	35	23	51	22	14	33	13	8	21	3160	1998	4483	1969	1201	2953	1191	704	1872
**Injuries**	16,176	14,784	17,140	9942	9402	10,400	6234	5328	6871	416,154	398,959	432,355	299,246	287,986	310,648	116,908	107,579	124,478
Transport injuries	3158	2985	3320	2395	2223	2535	763	697	824	118,835	112,149	125,027	93,880	87,222	99,215	24,955	23,296	26,816
Road injuries	2570	2407	2718	1900	1750	2023	670	608	729	96,237	90,168	101,922	74,795	68,816	79,808	21,442	19,870	23,284
Other transport injuries	587	546	629	494	459	531	93	86	100	22,597	21,037	24,197	19,084	17,703	20,506	3513	3247	3802
Unintentional injuries	8918	7810	9686	4484	4126	4784	4434	3626	4994	153,604	142,261	162,275	97,217	92,508	102,353	56,387	49,023	61,866
Falls	4622	3952	5128	2267	2040	2492	2355	1892	2751	71,048	65,105	76,431	44,015	40,775	47,662	27,033	22,935	30,494
Drowning	425	397	452	336	315	355	89	80	97	14,998	14,033	15,951	12,256	11,491	13,023	2742	2518	2990
Fire, heat, and hot substances	239	212	260	127	117	137	112	93	125	5023	4637	5389	3214	2970	3438	1809	1632	1973
Poisonings	108	98	117	67	61	73	41	36	45	3341	3059	3618	2282	2073	2496	1059	968	1153
Exposure to mechanical forces	243	221	262	181	165	196	62	54	69	7976	7324	8627	6524	5956	7089	1452	1322	1592
Adverse effects of medical treatment	739	635	899	329	282	391	410	331	534	12,712	11,238	15,377	6542	5645	7954	6170	5222	8162
Animal contact	25	22	28	19	17	22	6	5	7	767	687	862	596	520	690	171	156	189
Foreign body	2369	1997	2620	1045	932	1143	1324	1056	1492	32,696	29,539	35,138	17,490	16,260	18,685	15,206	13,011	16,710
Other unintentional injuries	90	83	99	77	71	85	13	11	15	3803	3465	4169	3405	3098	3749	398	360	440
Self-harm and interpersonal violence	4099	3877	4335	3062	2878	3273	1037	953	1120	143,716	136,336	151,705	108,149	101,572	115,116	35,567	33,153	38,273
Self-harm	3760	3539	3989	2839	2656	3043	921	843	1000	129,481	122,233	137,309	98,612	92,122	105,582	30,869	28,409	33,450
Interpersonal violence	335	314	358	221	207	236	114	106	123	13,975	13,062	14,942	9399	8775	10,079	4576	4255	4949

Following NCDs, 4.2% of deaths in 2019 were caused by CMNNDs (18,134, UI: 15,251–20,376) and 3.8% by injuries (16,176, UI: 14,784–17,140). Among CMNNDs, respiratory infections and tuberculosis, diarrheal diseases, and other infectious diseases accounted for 14,583 (UI: 12,105–16,536), 1213 (UI: 953–1485), and 647 deaths (UI: 562–796), respectively. HIV/AIDS accounted for 679 deaths (UI: 640–718). Of deaths related to injuries, the top three causes were falls at 4621 deaths (UI: 3952–5128), self-harm at 3759 (UI: 3539–3989), and road injuries at 2570 (UI: 2407–2718) deaths.

Total YLLs in 2019 were 6,330,140 (UI: 6,205,667–6,464,235); 3,644,301 in males and 2,685,839 in females. Similar to causes of death totals, 89.2% of YLLs were due to NCDs (5,643,796, UI: 5,530,194–5,762,752), followed by 6.6% due to injuries (416,154, UI: 398,959–432,355), and 4.3% due to CMNNDs (270,190, UI: 241, 863–293,260). The YLLs caused by injury disproportionately affected males at 299,246 YLLs, compared to 116,908 among females.

**Figure 2:**
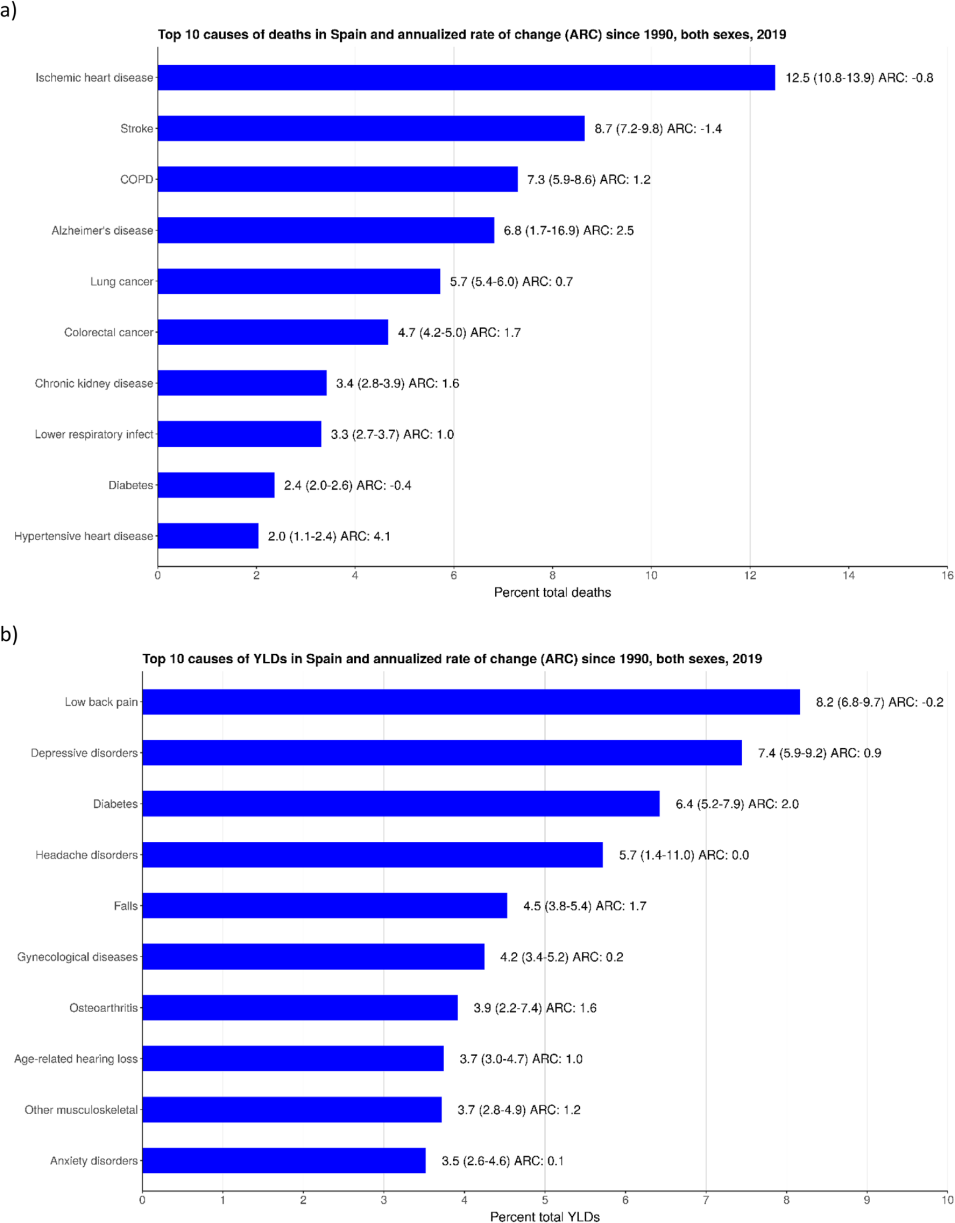

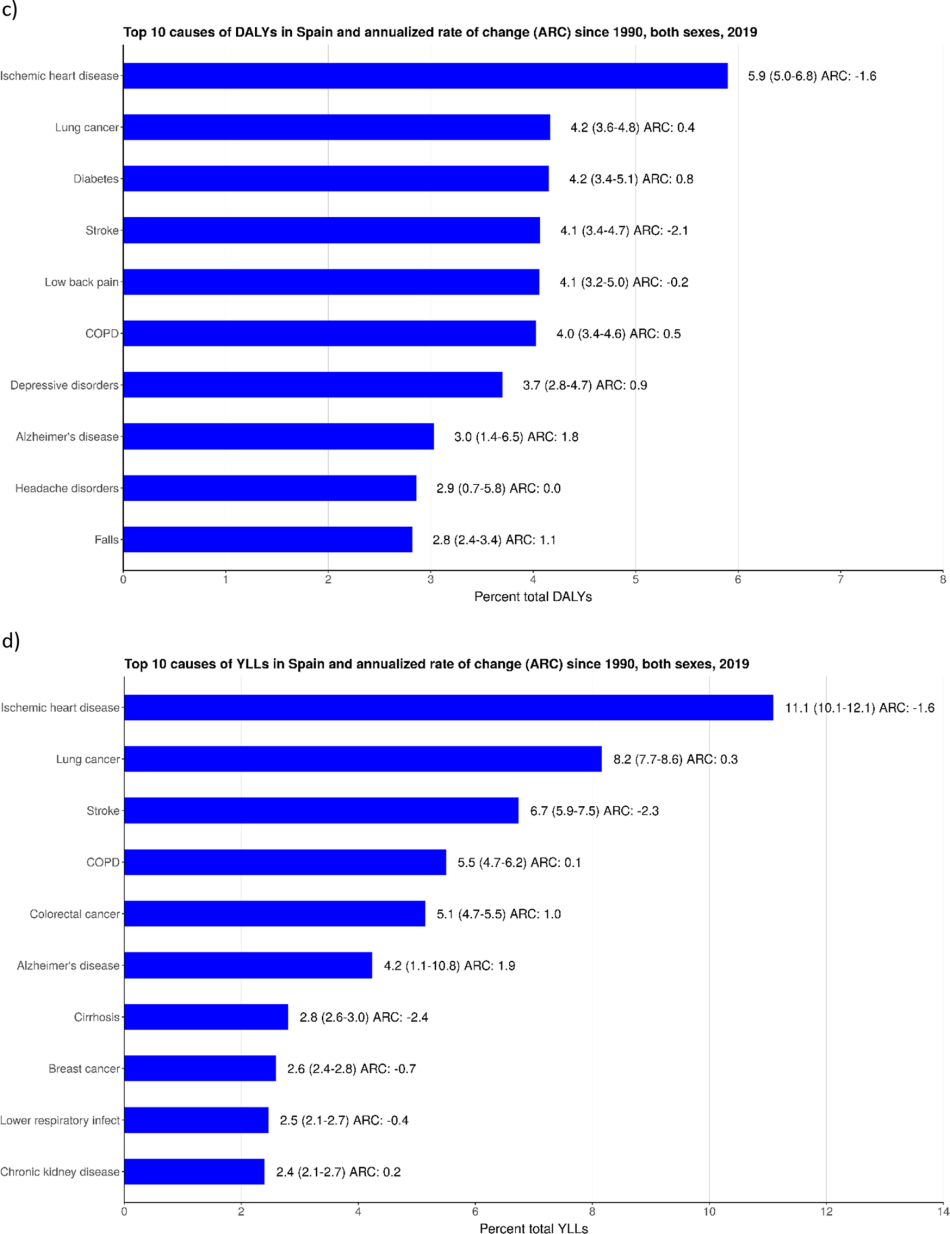
Top 10 causes of: (**a**) deaths; (**b**) years lived with disability (YLDs); (**c**) disability-adjusted life years (DALYs); and d) years of life lost (YLLs) in Spain and ARC (%), 2019. COPD, chronic obstructive pulmonary disease.

In 2019, IHD accounted for 12.5% (UI: 10.8–13.9) of all deaths (53,632, UI: 46,434–59,832), with an annual rate of change (ARC) of -0.8% from 1990; stroke accounted for 8.7% (UI: 7.2–9.8) of all deaths (37,092, UI: 30,981–42,048), with an ARC of -1.4%; and COPD accounted for 7.3% (UI: 5.9–8.6) of all deaths (31, 246, UI: 25,155–36,629), with an ARC of 1.2% (Fig. 2a, UI and changes since 1990 not shown in figure). Of the total YLDs, low back pain attributed to 8.2% (UI: 6.8–9.7), with an ARC of -0.2% (1121.0 YLDs per 100,000, UI: 806.2–1527.9), depressive disorders attributed to 7.4% (UI: 5.9–9.2), with an ARC of 0.9% (1021.5 YLDs per 100,000, UI: 724.4–1392.7), and diabetes attributed to 6.4% (UI: 5.2–7.9), with an ARC of 2.0% (885.2 YLDs per 100,000, UI: 572.5–1247.5) (Fig. 2b). Similar to deaths and YLDs, the top contributors to DALYs include IHD, which contributed to 5.9% (UI: 5.0–6.8) and had an ARC of -1.6% (1617 DALYs per 100,000, UI: 1474–1755), diabetes, which contributed to 4.2% (UI: 3.4–5.1) and had an ARC of 0.8% (1146 DALYs per 100,000, UI 842–1514), and lung cancer, which contributed to 4.2% (UI: 3.6–4.8) and had an ARC of 0.4% (1,139 DALYs per 100,000, UI: 1066–1202) (see Fig. 2c). The top contributors to YLLs also include IHD at 11.1% (UI: 10.1–12.1), with an ARC of -1.6% (702,210 YLLs, UI: 638,688–765,172); lung cancer at 8.2% (UI: 7.7–8.6), with an ARC of 0.3% (516,790 YLLs, UI: 484,690–545,363); and stroke at 6.7% (UI: 5.9–7.5), with an ARC of -2.3% (426,742 YLLs, UI: 371,628–473.043; Table 1, Fig. 2d).

IHD, stroke, and COPD were the three leading causes of death in Spain in both 1990 and 2019. In 2019, IHD caused 116.5 (UI: 100.9–130.0) deaths per 100,000 and stroke caused 80.6 (UI: 67.3–91.4) deaths per 100,000. While IHD and stroke related deaths decreased from 1990 to 2019, COPD-related deaths increased from 47.7 (UI: 43.4–50.9) to 67.9 (UI: 54.7–79.6) per 100,000, between 1990 and 2019. The main causes of death remained relatively similar from 1990 to 2019, with the exception of Alzheimer’s disease and lung cancer switching places as the fourth and fifth causes of death in 2019, compared to 1990 (Supp Fig. S3).

The leading conditions causing YLDs in Spain in 2019 were low back pain (1121.0, UI: 806.2–1527.9), depressive disorders (1021.5, UI: 724.4–1392.7), and diabetes (885.2, UI: 572.5–1247.5). Diabetes moved up from sixth position in 1990 and displaced headache disorders (792.8, UI: 189.2–1692.9), which covers the fourth position in 2019. Falls increased from eighth in 1990 to fifth in 2019, with 623.4 YLDs (433.2–882.7) per 100,000 (Supp Fig. S4).

IHD was the leading cause of DALYs in both 1999 and 2019. In 2019, IHD contributed to 1613.6 (UI: 1474.9–1755.4) DALYs per 100,000. In order of ranking, diabetes (1145.5, UI: 842.3–1513.6), lung cancer (1139.2, UI: 1065.8–1202.2), low back pain (1121.1, UI: 806.2–1527.9), and stroke (1113.4, UI: 989.8–1221.6) were the top five causes of DALYs in 2019 (see Supp Fig. S5).

For males, leading causes of DALYs were, in descending order, IHD (2014.8, UI: 1896.8–2148.0), lung cancer (1822.7, UI: 1695.5–1936.0), and COPD (1461.6, UI: 1284.7–1640.5) (see Supp Fig. S6a). In contrast, for females, leading causes of DALYs were low back pain (1368.4, UI: 980.4–1864.3), depressive disorders (1356.0, UI: 959.1–1835.0), and IHD (1229.0, UI:1051.3–1400.3) (see Supp Fig. S6b).

The same top six conditions contribute to DALYs in Spain, compared to other high-income countries, with IHD as the number one contributor to DALYs (see Supp Fig. S7). Globally, IHD ranks second as contributor to DALYs, while overall CMNNDs primarily contribute to DALYs.

Similar to DALYs, IHD has been the leading contributor to YLLs in Spain since 1990. Currently, IHD contributes to 1525.8 (UI: 1387.8–1662.7) YLLs per 100,000, followed by lung cancer with 1122.9 (1053.2–1185.0), which rose from fourth place in 1990, and stroke with 927.3 (UI: 807.5–1027.9), which dropped from second place in 1990 to third in 2019. Road injuries dropped from the third to the seventeenth position from 1990 to 2019, causing 209.1 (UI: 195.9–221.5) YLLs per 100,000 (Supp Fig. S8).

When YLLs are disaggregated by sex, results remain similar (see Supp Fig. S9a,b). IHD was the leading cause for both sexes; 1914.5 (UI: 1800.9–2042.0) for males and 1153.2 (UI: 970.7–1317.9) for females. For males, the next leading causes, in descending order, were lung cancer (1796.1, UI: 1672.6–1907.2), COPD (1050.4, UI: 908.4–1189.7), stroke (886.3, UI: 801.6–970.6), and colorectal cancer (865.4, 792.4–932.2). For females, the next leading causes, in descending order, were stroke (966.5, UI: 797.5–1112.8), Alzheimer’s disease (782.9, UI: 203.4–1938.7), breast cancer (689.4, 632.2–738.3), and colorectal cancer (555.8, UI: 490.8–607.5).

### Risk factors

**Figure 3:**
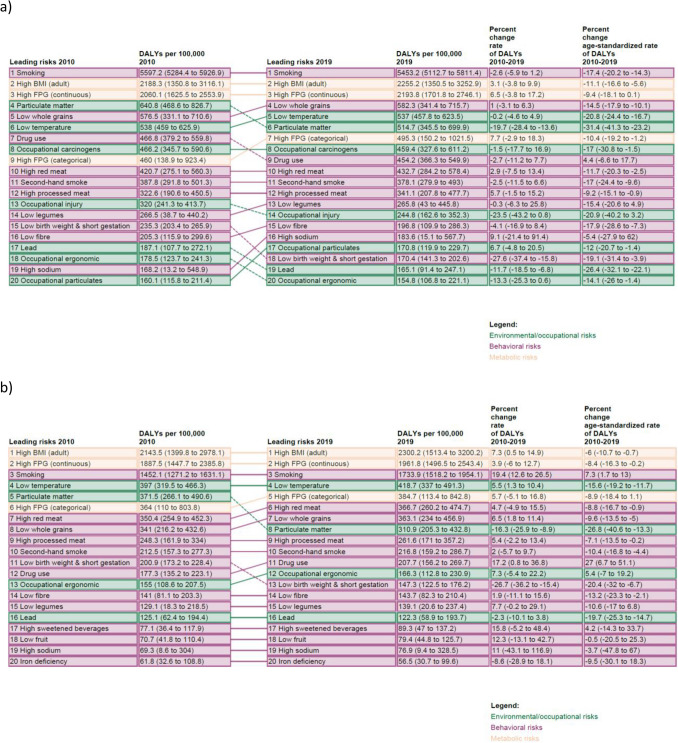
Changes in the rank of risk factors from 2010 to 2019 attributable to disability-adjusted life years (DALYs) in: (**a**) males; and (**b**) females. BMI, body mass index; FPG, fasting plasma glucose.

For males, smoking consistently ranked as the top risk factor for 2010 and 2019, attributable to DALYs (5453.2, UI: 5112.7–5811.4). High body mass index (BMI) (2255.2, UI: 1350.5–3252.9) and high fasting blood glucose (FPG) (2193.8, UI: 1701.8–2746.1) were the second and third ranked risk factors in both 2010 and 2019, for males (Fig. 3a). For females, in both 2010 and 2019, smoking (1733.9, UI: 1518.2–1954.1) ranked third, while high BMI (2300.2, UI: 1513.4–3200.2) and high FPG (1961.8, UI: 1496.5–2543.4) ranked first and second, respectively (Fig. 3b). For both sexes, a plurality of the top twenty risk factors were related to poor diet, and the new risk factor, low non-optimal ambient temperature, was among the top five risks in 2019.

The leading YLD risks in 2019 were, in descending order, high FPG, causing 987.4 YLDs (UI: 660.5–1372.4), high BMI, 955.8 (UI: 565.2–1470.0), and smoking, 838.8 (UI: 618.4–1080.9), each of which, similar to DALYs, have remained the three leading risks since 2010 (Supp Fig. S10).

For males, the leading risks for YLDs mirrored DALYs in 2019. These were, in descending order, smoking (1021.8, UI: 768.5–1300.5), high FPG (1006.9, UI: 671.8–1401.9), and high BMI (850.2, UI: 474.3–1344.3) (see Supp Fig. S11). In comparison, leading risks in females for YLDs were, in descending order, high BMI (1057.1, UI: 634.1–1576.9), high FPG (968.8, UI: 643.8–1350.2), and smoking (663.3, UI: 476.2–875.8) (see Supp Fig. S11b).

### Sustainable Development Goals

**Figure 4:**
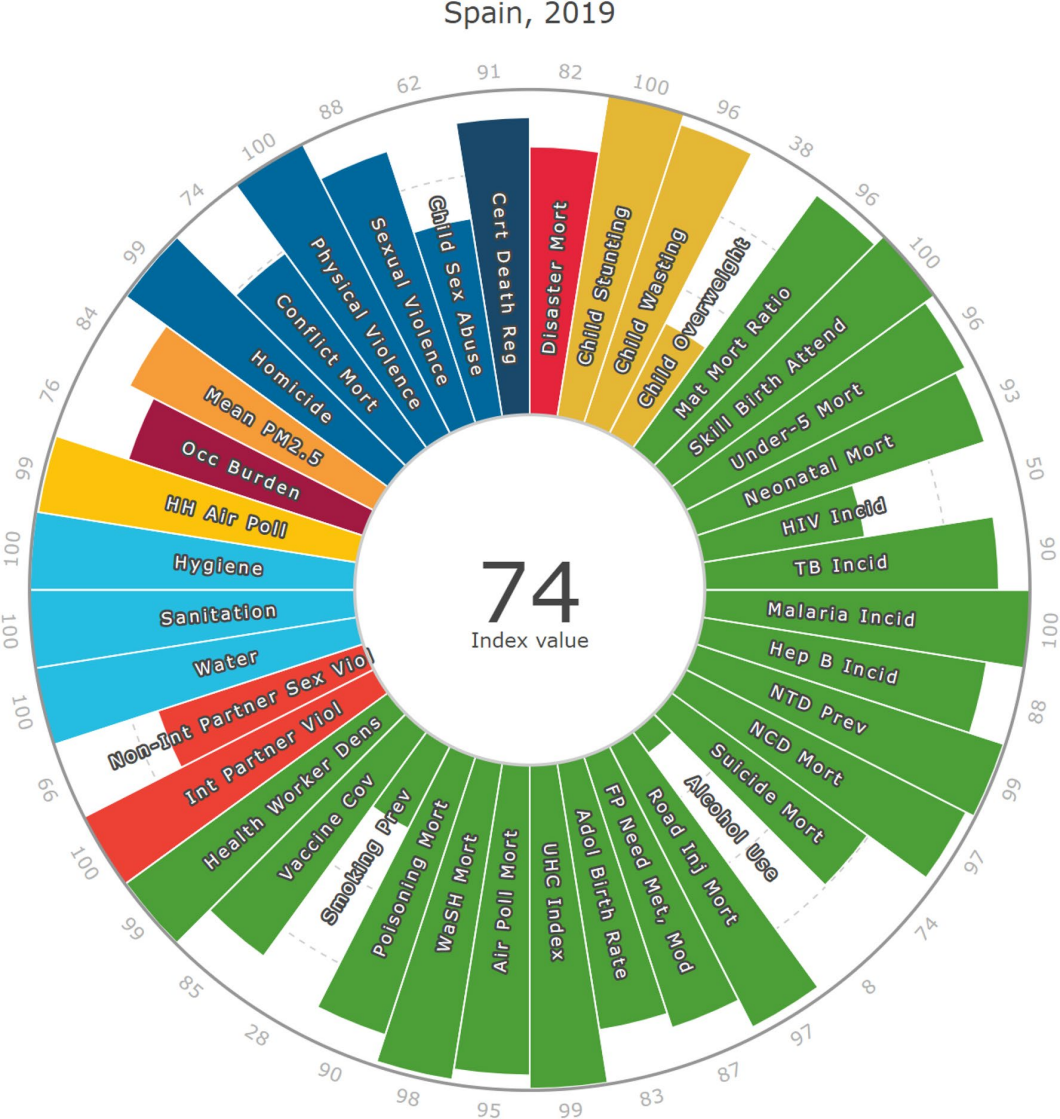
Sustainable Development Goal health-related index score components for Spain in 2019. Adol, adolescent; Attend, attendance; Cert, certified; Cov, coverage; Dens, density; FP, family planning; Hep, hepatitis; HH, household; Incid, incidence; Inj, injury; Int, intimate; Mat, maternal; Mod, modern contraception methods; Mort, mortality; NCD, non-communicable disease; NTD, neglected tropical disease; Occ, occupational; Prev, prevalence; Poll, pollution; Reg, registration; Skill, skilled; TB, tuberculosis; UHC, universal health coverage; Vio(l), violence; WASH, water, sanitation, and hygiene.

**Figure 5:**
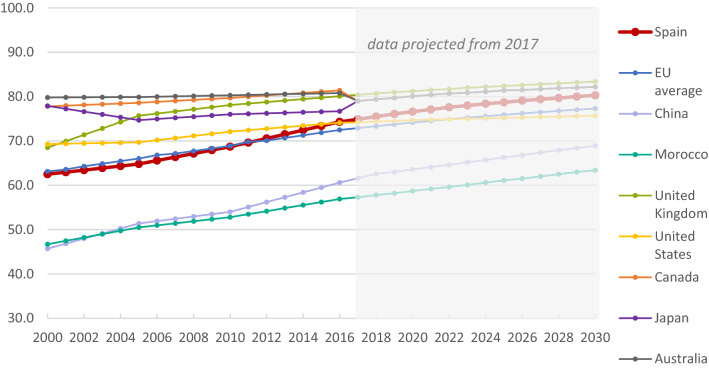
Trends in the Sustainable Development Goal health-related index score for Spain and comparator geographies from 2000 to 2017, with projections to 2030. EU, European Union.

The SDG health-related indicators for Spain result in an overall index score of 74 (Fig. 4), similar to Japan (76), the United States (75), and the European Union (EU) (74) (Fig. 5). Spain ranks 20 out of 195 countries and territories included in the index. It also achieved its highest performance (100) in hygiene, sanitation, intimate partner violence, skilled birth attendance, child stunting, and physical violence. Spain performed lowest in alcohol use (8), smoking prevalence (28), child overweight (38), and HIV incidence (50).

The health-related index score for Spain is projected to reach 80 by 2030, outpacing Japan (77), the United States (76), and the EU (77; Fig. 5). Although, it is projected that indicators for alcohol use (12), child overweight (32), smoking prevalence (36), and child sex abuse (59) will remain poor in 2030.

## Discussion

This study presents internationally comparable estimates of mortality, morbidity, and their risk factors in Spain and is the only study that has produced these estimates, with life expectancy being reported in this study and by the INE in Spain*.* GBD estimates for life expectancy are slightly higher compared to INE, as women were expected to live up to 86.2 years and men were expected to live up to 80.8 years; both sexes were expected to live, on average, 83.6 years.

The GBD 2019 study confirmed that NCDs, in particular IHD and cancers, are the largest contributors to morbidity and mortality in Spain. These results are similar to neighbouring European countries^10,20,21^. Musculoskeletal pathology, specifically low back pain and depression, also considerably contribute to the burden of disease, especially for women. These results highlight the influence of sedentary lifestyles^22,23^ and population ageing on the disease burden in Spain, the latter partially a positive consequence of the long-term benefits associated with improvements to the built environment that foster more physical activity^24^, advancement in occupational health and safety^25^, increases in educational attainment^26^, and universal healthcare^1^. In particular, in its 2018 review of SDG processes in Spain, the national government highlighted the importance of population-wide free-of-charge universal access to the health system, so as to enable attainment of the health targets under SDG 3: “Ensure healthy lives and promote well-being for all at all ages”^27^. In 2021, Spain formally renewed its commitment to the SDGs and issued a detailed 350-page report identifying 8 major challenges, of which health and public health are cross-cutting themes^28^. Several of the authors of this article contributed to this report, based on preliminary findings. Operationally, concrete actions to reach the SDGs will be largely within the purview of the 17 regions.

Like much of Europe, Spain has experienced rapid levels of population ageing due to increases in life expectancy and decreases in mortality and fertility since the mid-1990s^29^. Addressing population ageing requires a focus on health promotion and elderly care through strengthening long-term care facilities, social support services, and telehealth. In particular, monitoring of quality of life, functionality, and multimorbidity is even more important as the population ages^30,31^. Social protection benefits, such as pensions or sick leave, are key public health interventions that can help to offset cost-related issues of population ageing^32^. However, it is important to note that these policies may not reach those employed outside of the formal employment system. The influence of the Mediterranean diet has offered protective health benefits for aging populations, including in Spain^33–36^. However, such benefits are threatened by results in 2019, such as high FPG and high BMI, which are risk factors for cardiovascular^37^ and metabolic diseases such as diabetes^38^, which are among the top 10 causes of death.

Additionally, targeted approaches in national planning and the decentralised subnational service delivery of healthcare services should address the burden of other NCDs such as IHD, low back pain, and depressive disorders, which drive DALYs and YLDs. Consistent with our results, recent research in Spain identified a disproportionate burden of low back pain among women compared to men^39^, while earlier studies identified the reverse relationship^40,41^. Low back pain significantly impacts economic productivity and worker health^42^, and is important to address through occupational health interventions^43^ in addition to health services, which are already well-used^39^. In contrast, mental health services are underutilised in Spain^44^, possibly indicating problems with access to such services. Improving mental health services is also challenged by a lack of coordination across regions and sectors in the past decade^45,46^. Symptoms of depression and other mental health issues, which are disproportionately experienced by women^47–49^ and vulnerable groups^50,51^, have become more prevalent^52^ and are most associated with lower education and income^49^. Gender inequalities in the diagnoses of mental health disorders may be attributed to socio-cultural factors. Notably, the pathologisation of “feminine attributes”, such as emotional expression, may lead women to be more diagnosed with mental health problems than men, who are more likely to conceal their emotions^49^. Further intersecting with gender is age and social vulnerability, whereby older and other vulnerable patients, viewed as less resilient to suffering, are more likely to be diagnosed with mental health problems. This issue should also be addressed through a public health approach, recognising the overlapping and intersecting nature of the social determinants of health^2^.

This study’s results show that some behavioural and metabolic factors, such as smoking, diet, BMI, and FPG contribute heavily to the burden of disease in Spain, much like neighbouring European countries^20^. This must be adequately addressed by public health approaches that address population risk factors. For example, public policy should consider obesogenic environments that contribute to sedentary lifestyles and should be re-designed using a public health approach by, for instance, encouraging regular physical activity, healthy eating, and smoking cessation and prevention^2,53–56^. Improving primary care services will enable better implementation of interventions to promote behavioural changes related to health habits and mental health promotion. For example, the incorporation of behavioural health and quality of life or well-being tools with patient-reported outcomes into primary care delivery can improve system diagnostic and referral capacities^57,58^. Furthermore, anti-smoking legislation, such as removing tobacco vending machines, should be strengthened to address child and adolescent smoking, particularly in males^59,60^, and to better align with the WHO Framework Convention on Tobacco Control, of which Spain is a signatory^61^.

Policymakers must also consider non-optimal ambient temperatures, which, despite decreases in recent years of low-temperature related mortality^62^, have significantly contributed to morbidity in Spain. It might be useful to extend the National Plan for Preventive Actions Against the Health Effects of Excess Temperatures, to implement both low temperature and heat adaptation strategies sub-nationally, which have demonstrated effectiveness in other high-income countries^63^. In addition to warmer temperatures, which are projected to increase, policymakers must also consider sub-national approaches to address the continued impacts of low temperatures, which may contribute to higher mortality among vulnerable groups in some regions of in Spain^64,65^.

Between March and May 2020, COVID-19 ranked as the leading cause of mortality in Spain^66^ and in November 2022 Spain continued to rank among the first dozen countries in total number of confirmed cases^67^. The pandemic has led to a healthcare provision crisis that greatly decreased access to many routine health services and, during peak waves, access to critical equipment such as intensive care unit beds and artificial ventilators. The downstream effects of unattended acute and chronic conditions^68^, especially mental health problems, will exacerbate morbidity and mortality projections in Spain. Multiple sets of concrete recommendations to manage COVID-19 have been issued targeting Spain specifically^69^, and in a recent global Delphi study, co-led by Spanish authors^70^. However, a major challenge in Spain remains to be coordination among the 17 regions and the national government. Our study provides the 2019 results of disease burden and risk factors in Spain, so that health researchers and decision makers have a pre-pandemic baseline to compare pandemic findings to.

### Future avenues

While it is important to understand mortality and morbidity and their drivers, this data is insufficient to inform adequate public health interventions. Further studies, based on the results presented, are necessary to create appropriate and equitable evidence-based interventions for public health. Future research must focus on health equity within Spain that, in addition to sex and age disparities, investigates the drivers of disparities among vulnerable groups, who may be disproportionately impacted by policies. Spain should strive to ensure that standardised data at the regional level is available to inform research and decision making, and, where possible, include disaggregated data by specific vulnerable groups, such as migrants and people experiencing homelessness, as well as different occupational categories. GBD should endeavour to assess mortality, morbidity, and risk by these populations as well. Future studies should consider examining further lifestyle and behavioural factors that contribute significantly to morbidity and mortality in Spain, especially smoking, alcohol use, and sedentary lifestyles. Considering the 2008 financial crisis and the ongoing COVID-19 pandemic, future research should aim to examine how these experiences have influenced and will continue to impact on the health trajectory of Spain, including in each of the 17 regions, especially in terms of mental health and access to care more broadly. In particular, the at least eight waves of the COVID-19 pandemic experienced to date in Spain, and any subsequent ones, will require a paradigm shift. A change in care models in primary care services and selected hospital services is envisaged, given the expected high frequency of Long COVID patients and all sequelae of COVID-19, which will require care and attention within often limited available resources^71^. Finally, the relatively low burden of infectious diseases in Spain has led to their de-prioritisation in public health research focused on Spain. This field would benefit from an analysis of infectious diseases pre-, during, and after the pandemic and in relation to changing trends in ambient temperatures.

### Strengths and limitations

The primary limitation of the GBD Study is the availability of primary data. In the case of Spain, coding was performed by Spain’s INE*,* the most important data source for GBD estimates for Spain, and is considered among the best in terms of validity and completeness of data within WHO’s European Region. Despite this, there might be difficulties representing the full uncertainty around estimates due to discrepancies in coding and definitions between INE and GBD. However, this study did not compare GBD estimates for Spain with data from Spain. Detailed explanations of the limitations of each specific model in GBD 2019 are reported elsewhere^57^.

Additionally, GBD data are available at the subnational level for 22 countries but not for Spain, which should provide this information as well. Furthermore, Spain does not have its own disability weights, and these are derived from studies in other countries, which challenges the accuracy of these results.

Regarding the SDG projections, a major strength is that it is a single and robust measurement that is useful for policymakers to interpret and compare the performance on all health-related SDG measures. Moreover, it can help to better understand progress overtime for all indicators. However, data sparsity and variations in case-definitions may lead to underreporting and uncertainty of particular outcomes. Furthermore, the COVID-19 pandemic will have likely affected several health dimensions in 2020 and 2021, and possibly beyond. However, the research presented in this study establishes a unique baseline for future research on the impact of the COVID-19 pandemic.

## Conclusion

Similar to recent analyses of the burden of disease in Spain, non-communicable diseases, particularly cardiovascular diseases, continued to be the predominant cause of morbidity and mortality in 2019. Behavioural risks, such as smoking and poor diet, and environmental risks, such as non-optimal temperatures, contributed substantially to the disease burden, indicating focus areas for prevention for health authorities. The health system should also address the consequences of population ageing, such as morbidity from musculoskeletal conditions and Alzheimer’s disease, in addition to the long-term impacts of the COVID-19 pandemic, including Long COVID, which threaten health-related SDG progress.

## Supplementary Information

Supplementary Information.

## Data Availability

To download the data used in these analyses, please visit the Global Health Data Exchange GBD 2019 website (http://ghdx.healthdata.org/gbd-2019).
