# Supplementary material for: A GBD 2019 study of health and Sustainable Development Goal gains and forecasts to 2030 in Spain
Source: Sci Rep. 2022 Dec 7;12:21154. doi: 10.1038/s41598-022-24719-z (PMC9729199; doi:10.1038/s41598-022-24719-z)
Supplement: Supplementary file 1 — Supplementary Information. [file 41598_2022_24719_MOESM1_ESM.pdf]

A GBD 2019 study of health and Sustainable Development Goal gains and forecasts to 2030 in Spain

Authors: Jeffrey V Lazarus, PhD[1,2*], Alberto Ortiz[3,4], Stefanos Tyrovolas[5,6], Esteve Fernández[7,8], Danielle Guy, MSc[1], Trenton M White[1], MPH, Rui Ma, MSc (IHME)[9], Simon I Hay, DSc (IHME)[9,10], Mohsen Naghavi, PhD (IHME)[9,10], Joan B Soriano, MD[11,12], the GBD 2019 Spain Collaborators

Supplemental material

Appendix 1. List of publications using GBD methodology and/or data in Spain
Appendix 2. Methodological supplement
Supp Figure 1. Age and sex pyramid of the Spanish population (millions) in: a) 2019; and b) 2030
Supp Figure 2. Life expectancy in Spain from 1990–2019
Supp Figure 3. Changes in the rank of the causes of death (rate per 100,000) from 1990–2019
Supp Figure 4. Changes in the rank of the causes of years lived with disability (YLDs) (rate per 100,000) from 2010–2019
Supp Figure 5. Changes in the rank of causes of disability-adjusted life years (DALYs; rate per 100,000) from 2010–2019
Supp Figure 6. Ranking in the causes of disability-adjusted life years (DALYs; rate per 100,000) in 2019 in: a) males; and b) females
Supp Figure 7. Ranking in the causes of disability-adjusted life years (DALYs; rate per 100,000) in all ages in 2019 in: a) Spain; b) high-income countries; and c) the world
Supp Figure 8. Changes in the rank of the causes of years of life lost (YLLs) (rate per 100,000) from 1990–2019
Supp Figure 9. Ranking in the causes of years of life lost (YLLs; rate per 100,000) in: a) males; and b) females
Supp Figure 10. Changes in the rank of the risk factors for years lived with disability (YLDs; rate per 100,000) from 2010–2019
Supp Figure 11. Changes in the rank of risk factors for years lived with disability (YLDs; rate per 100,000) in 2019 in: a) males; and b) females

Appendix 1. List of publications using GBD methodology and/or data in Spain

1. Ortiz A, Sanchez-Niño MD, Crespo-Barrio M, et al. The Spanish Society of Nephrology (SENEFRO) commentary to the Spain GBD 2016 report: Keeping chronic kidney disease out of sight of health authorities will only magnify the problem. Nefrología. 2019 Jan;39(1):29–34.
2. Soriano JB, Rojas-Rueda D, Alonso J, et al. The burden of disease in Spain: Results from the Global Burden of Disease 2016. Med Clin (Barc). 2018 Sep 14;151(5):171–90.
3. Mueller N, Rojas-Rueda D, Basagaña X, et al. Health impacts related to urban and transport planning: A burden of disease assessment. Environ Int. 2017;107:243–57.
4. Fernández de Larrea-Baz N, Morant-Ginestar C, Catalá-López F, Gènova-Maleras R, Álvarez-Martín E. Disability-adjusted Life Years Lost to Ischemic Heart Disease in Spain. Rev Española Cardiol. 2015 Nov;68(11):968–75.
5. Lara E, Garin N, Ferrari AJ, et al. The Spanish Burden of Disease 2010: Neurological, mental and substance use disorders. Rev Psiquiatr y Salud Ment. 2015 Oct 1;8(4):207–17.
6. Catalá-López F, De Larrea-Baz NF, Morant-Ginestar C, Álvarez-Martín E, Díaz-Guzmán J, Génova-Maleras R. The national burden of cerebrovascular diseases in Spain: A population-based study using disability-adjusted life years. Med Clin (Barc). 2015;144(8):353–9.
7. Haro MM, Tyrovolas S, Garin N, et al. The burden of disease in Spain: Results from the global burden of disease study 2010. BMC Med. 2014 Dec 5;12(1).
8. Catalá-López F, Gènova-Maleras R, Ridao M, et al. Burden of disease assessment with summary measures of population health for the Region of Valencia, Spain: A population-based study. Med Clin (Barc). 2013 Apr 20;140(8):343–50.
9. Gènova-Maleras R, Álvarez-Martín E, Morant-Ginestar C, Fernández de Larrea-Baz N, Catalá-López F. Measuring the burden of disease and injury in Spain using disability-adjusted life years: An updated and policy-oriented overview. Public Health. 2012;126(12):1024–31.
10. Sánchez-Valle E, Posada M, Villaverde-Hueso A, et al. Estimating the burden of disease for autism spectrum disorders in Spain in 2003. J Autism Dev Disord. 2008 Feb;38(2):288–96.
11. Villaverde-Hueso A, Sanchez-Valle E, Morant C, et al. Estimating the Burden of Scleroderma Disease in Spain. J Rheumatol [Internet]. 2007 [cited 2020 Mar 23];34(11):2236–42. Available from: https://pubmed.ncbi.nlm.nih.gov/17918783/
12. Ayuso-Mateos JL, Gutierrez-Recacha P, Haro JM, Chisholm D. Estimating the prevalence of schizophrenia in Spain using a disease model. Schizophr Res. 2006 Sep;86(1–3):194–201.
13. Cortés M, Pereira J, Peña-Rey I, Génova R, Amela C. Disease burden due to vaccinable diseases in the Spanish population aged less than 15 years old. Gac Sanit. 2004 Jan 1;18(4):312–20.

Appendix 2. Methodological supplement

1. ***Official projection of the population pyramids and of life expectancy up to 2100***

IHME forecasting and life expectancy estimates are created by modeling the future population in reference and alternative scenarios. These are modeled as functions of fertility, migration, and mortality rates. First, statistical models were developed for completed cohort fertility at 50 years old (CC50). This indicator is used because it is more stable over time compared to the total fertility rate. CCF50 was then modelled as a time-series random walk function taking into account educational attainment as well as contraceptive met need. CC50 and covariates were then used to model age-specific fertility rates up to the year 2100. IHME considered underlying mortality, a risk factor scalar, and an autoregressive integrated moving average (ARIMA) model. Second, IHME modelled net migration as a function of the Socio-demographic Index, crude population growth rate, and deaths from war and natural disasters using the ARIMA model. IHME then developed a reference scenario and alternative scenarios based on contraceptive met need, educational attainment, and Spain's estimated gross domestic product. Past data inputs, model estimation, and forecast data distributions were used to develop forecast uncertainty intervals (UIs). Detailed techniques for forecasting methods are published elsewhere.[1]

2. ***Population, fertility, mortality, and migration estimates***

Data used to produce demographic assessments of the key indicators of fertility, mortality, migration, and population in Spain are derived from the following sources:

- Vital registration data: live births and deaths, 1950– 2017
- Spain Population and Housing census data, INE: 1950–2017
- Spain deaths by cause, age, month, and sex, INE: 1980–2017
- UN Demographic Yearbook 1997–2017

All 2,360 original data records for Spain are available for download at: http://ghdx.healthdata.org/geography/spain.

These data sources are synthesized and corrected for known biases using the GBD spatiotemporal Gaussian process regression (ST-GPR). ST-GPR then generates age-specific fertility rates for 5-year age groups between ages 15 and 49 years. This was extended to estimate age-specific mortality for groups 10-14 and 50-54, which was then aggregated to create the total fertility rate from 10 and 54. ST-GPR also estimates adult mortality as the probability of death between ages 15 and 60 years based on vital registration, sample registration, and sibling histories. 1000 draw-level estimates were produced by the demographic estimation processes to estimate uncertainty.

Using a relational model life table system, IHME estimated HIV-free life tables using estimates of under-5 and adult mortality rates. A Bayesian hierarchical cohort component model analyzing estimated age-specific fertility and mortality rates were used to estimate annual and single-year age estimates of net migration and population. 1000 draw-level estimates were produced by the

demographic estimation processes to estimate uncertainty. Detailed methods are described elsewhere.[2]

3. SDG indicators and projections to 2030

GBD constructs national-level estimates for 41 health-related sustainable development goal (SDG) indicators from 1997-2017. For certain health-related SDGs, GBD also measures differences by sex and socio-demographic index quintile. The health-related SDG index is based on the value of each indicator, up to 100. For this, the value for each indicator was transformed on a scale of 0-100. In this scale, 0 indicates the 2.5th percentile and 100 indicates the 97.5th percentile of 1000 draws calculated from 1990 to 2030. Future estimates were developed through a forecasting framework based on the broader GBD study. This forecasting framework also used weighted averages of indicator-specific and country-specific annualized rates for change from 1990 to 2017. To assess the attainment of indicators, IHME first used mean values projected for 2030 and then used the probability of attainment in 2030, which was calculated from 1000 draws. IHME analyzed past trends to do a global attainment analysis on the feasibility of attaining SDG targets. The global annualized rates of change to meet SDG targets from 2015 to 2030 were calculated using 2015 averages of indicators with defined targets. Within the distribution of country-level rates of change from 1990-2015, IHME identified the percentile of the required global annualized rates of change. The past rate of change at the mean of these global percentile values across indicators was applied to all health-related SDG indicators. This was then used to estimate 2030 global average value and percent change over time for each indicator. New indicators added from GBD 2016 include health worker density, sexual violence by non-intimate partners, population census status, and prevalence of physical and sexual violence (reported separately). Detailed methods on SDG forecasting are available elsewhere.[3]

4. Mortality data

The Cause of Death Ensemble model and spatiotemporal Gaussian process regression were used to calculate cause-specific death rates and cause fractions. To match the total all-cause deaths calculated as part of the GBD population, fertility, and mortality estimates (see section 2 above), IHME adjusted cause-specific deaths. To calculate YLLs, deaths were multiplied by standard life expectancy at each age. To make sure there was consistency between incidence, prevalence, remission, excess mortality, and cause-specific mortality for most causes IHME used DisMod-MR 2.1, a Bayesian meta-regression modelling tool. To calculate YLDs, prevalence estimates were multiplied by disability weights for mutually exclusive sequelae of diseases and injuries.

Results were considered in the context of the Socio-demographic Index (SDI). The SDI is a composite indicator including income, education, and fertility rate in women younger than 25 years. For each metric, IHME developed uncertainty intervals using the 25th and 975th ordered 1000 draw values of the posterior distribution. IHME does not have estimates for 37 years of cause of death data by cause, sex, and age for Spain by the 16 regions. This is because Spain is not one of the locations for which subnational GBD data are produced.

GBD uses vital registration with medical certification of cause of death for cause of death analysis. The GBD cause of death list includes cause of death data obtained using various revisions of the International Classification of Diseases and Injuries (ICD). Deaths that could not be the underlying cause of death (e.g. cardiopulmonary failure) or were inadequately specified (e.g. injury from undetermined intent were reassigned to the most probable underlying causes of death. Deaths were redistributed based on evidence from published studies or expert judgment, or statistical algorithms.[4] Detailed methods for mortality data are published elsewhere.[5]

References

1 Vollset SE, Goren E, Yuan CW, et al. Fertility, mortality, migration, and population scenarios for 195 countries and territories from 2017 to 2100: a forecasting analysis for the Global Burden of Disease Study. *Lancet* 2020;**396**(10258):1285–306.

2 Abbafati C, Machado DB, Cislaghi B, et al. Global age-sex-specific fertility, mortality, healthy life expectancy (HALE), and population estimates in 204 countries and territories, 1950–2019: a comprehensive demographic analysis for the Global Burden of Disease Study 2019. *Lancet* 2020;**396**(10258):1160–203.

3 Lozano R, Fullman N, Abate D, et al. Measuring progress from 1990 to 2017 and projecting attainment to 2030 of the health-related Sustainable Development Goals for 195 countries and territories: a systematic analysis for the Global Burden of Disease Study 2017. *Lancet* 2018;**392**(10159):2091–138.

4 Institute for Health Metrics and Evaluation. Determining causes of death: How we reclassify miscoded deaths [Internet]. 2018 [cited 2020 Mar 25]. Available from: http://www.healthdata.org/acting-data/determining-causes-death-how-we-reclassify-miscoded-deaths

5 Abbafati C, Abbas KM, Abbasi-Kangevari M, et al. Global burden of 369 diseases and injuries in 204 countries and territories, 1990–2019: a systematic analysis for the Global Burden of Disease Study 2019. *Lancet* 2020;**396**(10258):1204–22.

Supp Figure 1. Age and sex pyramid of the Spanish population (millions) in: a) 2019; and b) 2030

a)

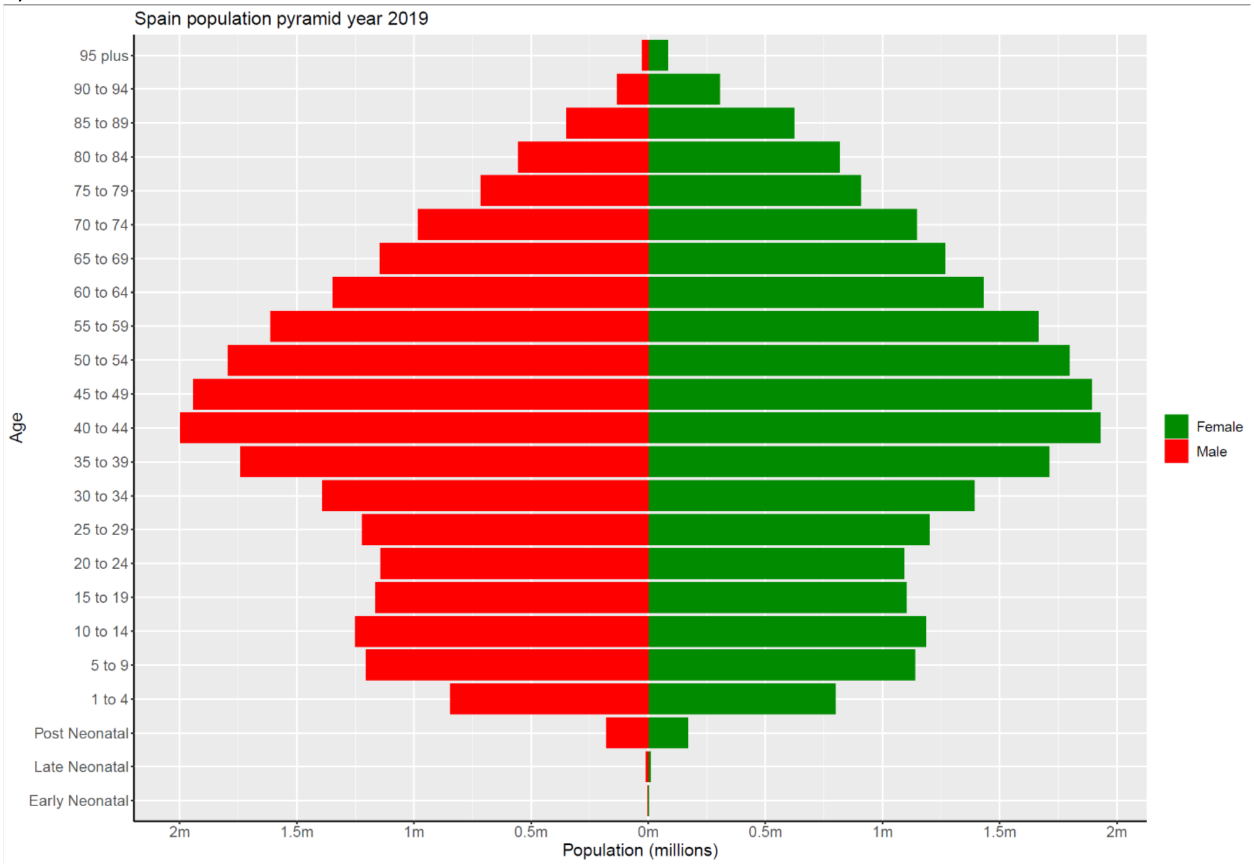

b)

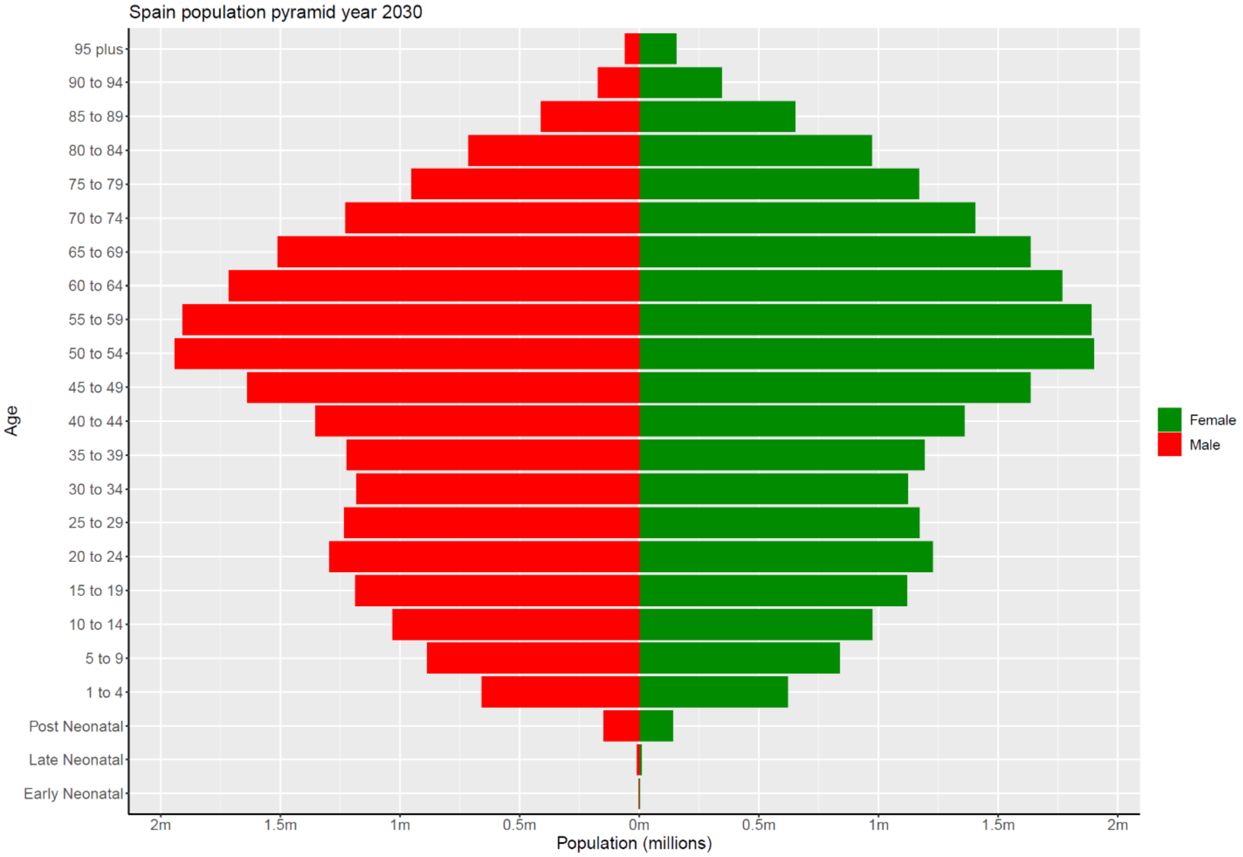
Spain population pyramid year 2030
95 plus
90 to 94
85 to 89
80 to 84
75 to 79
70 to 74
65 to 69
60 to 64
55 to 59
50 to 54
45 to 49
40 to 44
35 to 39
30 to 34
25 to 29
20 to 24
15 to 19
10 to 14
5 to 9
1 to 4
Post Neonatal
Late Neonatal
Early Neonatal
Age
2m
1.5m
1m
0.5m
0m
0.5m
1m
1.5m
2m
Population (millions)
Female
Male

Supp Figure 2. Life expectancy in Spain from 1990–2019

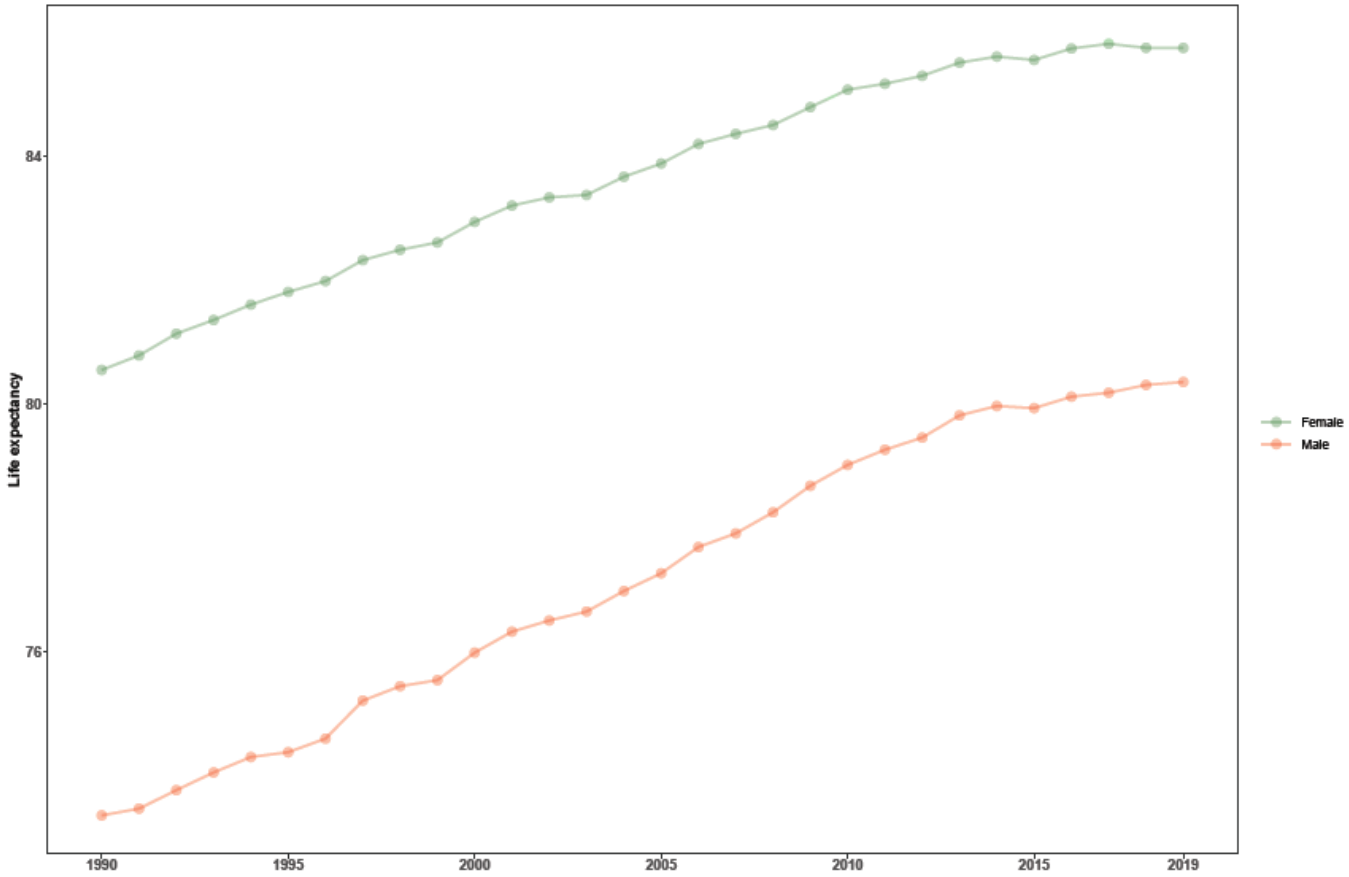

Supp Figure 3. Changes in the rank of the causes of death (rate per 100,000) from 1990–2019

Leading causes 1990	Deaths per 100,000 1990
1 Ischemic heart disease	148.7 (136.5 to 156.1)
2 Stroke	121.3 (109.8 to 128.9)
3 COPD	47.7 (43.4 to 50.9)
4 Lung cancer	42.9 (41.5 to 44.3)
5 Alzheimer's disease	30.6 (7.5 to 79.6)
6 Cirrhosis	29.7 (28.2 to 30.8)
7 Colorectal cancer	26.5 (25 to 27.7)
8 Diabetes	24.9 (22.7 to 26.3)
9 Road injuries	24.1 (23.4 to 24.6)
10 Lower respiratory infect	23.2 (21 to 24.7)
11 Stomach cancer	21.9 (20.6 to 22.8)
12 Chronic kidney disease	19.8 (17.8 to 21.4)
13 Breast cancer	16.8 (15.8 to 17.5)
14 Cardiomyopathy	13.7 (8.7 to 15.6)
15 Prostate cancer	13.7 (10.2 to 16.1)
16 Bladder cancer	10.5 (9.8 to 11)
17 Pancreatic cancer	9.9 (9.3 to 10.3)
18 Self-harm	9.1 (8.8 to 9.4)
19 Atrial fibrillation	7.8 (6.5 to 9.9)
20 Leukemia	7 (6.7 to 7.3)
22 Parkinson's disease	6.9 (6.3 to 7.3)
26 Hypertensive heart disease	5.9 (5 to 7.3)
27 Nonrheum valv diseases	5.8 (5.3 to 6.2)

Leading causes 2019	Deaths per 100,000 2019	Percent change rate of Deaths 1990-2019	Percent change age-standardized rate of Deaths 1990-2019
1 Ischemic heart disease	116.5 (100.9 to 130)	-21.6 (-27.4 to -14.7)	-59.5 (-61.6 to -56.5)
2 Stroke	80.6 (67.3 to 91.4)	-33.6 (-39.6 to -27)	-67.5 (-70.1 to -64.6)
3 COPD	67.9 (54.7 to 79.6)	42.2 (21.4 to 65.7)	-29.2 (-38.1 to -18.6)
4 Alzheimer's disease	63.5 (16.2 to 156.5)	107.3 (87 to 134.4)	-15.6 (-21 to -8)
5 Lung cancer	53.3 (49.4 to 56.4)	24.1 (15.3 to 31.8)	-12.8 (-18.4 to -7.6)
6 Colorectal cancer	43.5 (38.6 to 47.3)	64 (51.2 to 76.2)	-0.6 (-6.9 to 6.2)
7 Chronic kidney disease	31.8 (26.3 to 36.4)	60.8 (41.5 to 80.2)	-25.2 (-32.6 to -16.9)
8 Lower respiratory infect	30.8 (25.5 to 35.1)	32.6 (18.1 to 47.7)	-40.2 (-45.8 to -34.2)
9 Diabetes	22 (18.6 to 24.4)	-11.4 (-20.6 to -2.9)	-53.9 (-57.8 to -49.9)
10 Hypertensive heart disease	19 (9.8 to 22.6)	224 (54.4 to 288.1)	41.8 (-31.1 to 69)
11 Prostate cancer	18.3 (15.1 to 26.4)	33.5 (15.2 to 71.1)	-25.5 (-35.9 to -2.9)
12 Cirrhosis	17.9 (16.1 to 19.8)	-39.9 (-45.3 to -32.7)	-60.4 (-63.5 to -56.8)
13 Breast cancer	17.5 (15.4 to 19.2)	4.3 (-5.1 to 12.8)	-34.8 (-39.5 to -30.2)
14 Pancreatic cancer	17.2 (15.2 to 18.9)	73.9 (57.9 to 90.5)	14.8 (4.7 to 25.5)
15 Atrial fibrillation	16 (12.8 to 20.2)	106.3 (86.1 to 126)	-10.6 (-18.3 to -1.4)
16 Stomach cancer	15.7 (14 to 17.1)	-28.1 (-33.7 to -22.3)	-55.1 (-58 to -51.8)
17 Bladder cancer	14.3 (12.5 to 16.1)	36.6 (22.3 to 52.3)	-19.7 (-27.2 to -10.5)
18 Cardiomyopathy	14 (11.3 to 16.2)	1.9 (-12.3 to 43.5)	-48.6 (-55.1 to -28.1)
19 Nonrheum valv diseases	13.4 (11 to 15.6)	130.5 (100.9 to 161.9)	12.2 (-0.4 to 26.1)
20 Parkinson's disease	13.3 (11.6 to 14.4)	92.6 (79.6 to 105.2)	1.2 (-4.9 to 7.6)
24 Leukemia	9.1 (8.1 to 10.1)	30.1 (17.3 to 43.2)	-20.8 (-27.5 to -14)
25 Self-harm	8.2 (7.7 to 8.7)	-10.5 (-16.3 to -4)	-24.9 (-29.8 to -19.6)
36 Road injuries	5.6 (5.2 to 5.9)	-76.8 (-78.3 to -75.4)	-79.7 (-80.9 to -78.5)

Legend:
Communicable, maternal, neonatal and nutritional
Non-communicable
Injuries

Supp Figure 4. Changes in the rank of the causes of years lived with disability (YLDs) (rate per 100,000) from 1990–2019

Leading causes 1990	YLDs per 100,000 1990	Leading causes 2019	YLDs per 100,000 2019	Percent change rate of YLDs 1990-2019	Percent change age-standardized rate of YLDs 1990-2019
1 Low back pain	1170.9 (839.4 to 1557.2)	1 Low back pain	1121 (806.2 to 1527.9)	-4.3 (-13.2 to 6.5)	-18.3 (-26.1 to -10.1)
2 Depressive disorders	798.1 (559 to 1067.3)	2 Depressive disorders	1021.5 (724.4 to 1392.7)	28 (19.1 to 36.2)	18.6 (9.7 to 27.3)
3 Headache disorders	787.5 (162.9 to 1690.7)	3 Diabetes	885.2 (572.5 to 1247.5)	79.8 (64.6 to 95)	37.8 (26.7 to 49.4)
4 Gynecological diseases	554.5 (374.2 to 767.1)	4 Headache disorders	792.8 (189.2 to 1692.9)	0.7 (-4.1 to 17.2)	0.2 (-3.3 to 5)
5 Diabetes	492.2 (321.8 to 694.4)	5 Falls	623.4 (433.2 to 882.7)	62.2 (57.5 to 67.3)	29.6 (25.6 to 34.4)
6 Anxiety disorders	469.4 (318.9 to 662.5)	6 Gynecological diseases	584.1 (392.5 to 808.8)	5.3 (1 to 9.7)	-1.3 (-3.6 to 0.9)
7 Age-related hearing loss	386 (262.3 to 548.8)	7 Osteoarthritis	541.1 (259.5 to 1106.3)	58.6 (41.6 to 104.4)	15.3 (3.1 to 47.8)
8 Falls	384.4 (266 to 553.2)	8 Age-related hearing loss	514.4 (346.3 to 735.5)	33.3 (27.2 to 39.6)	-7.3 (-11.4 to -3.3)
9 Other musculoskeletal	359.2 (237.9 to 510.7)	9 Other musculoskeletal	508.8 (346.2 to 713.8)	41.6 (30.6 to 55.3)	21 (11.7 to 32)
10 Osteoarthritis	341.3 (173.2 to 677.8)	10 Anxiety disorders	482.6 (325.8 to 682.1)	2.8 (-6.7 to 12.7)	5.4 (-2.7 to 14)
11 Endo/metab/blood/immune	339.4 (227.8 to 473.3)	11 Endo/metab/blood/immune	396.3 (264 to 562.6)	16.8 (9.5 to 23.9)	-1 (-6.1 to 4)
12 Oral disorders	325.6 (204.7 to 471.5)	12 Blindness and vision loss	366.7 (259.3 to 495)	28.1 (23.1 to 33.5)	-6.8 (-10.4 to -3.4)
13 Neck pain	307.4 (205.4 to 442.4)	13 Neck pain	359.7 (240.3 to 522.5)	17 (10.2 to 22.7)	-0.2 (-2.7 to 2.4)
14 Blindness and vision loss	286.2 (202.5 to 386.9)	14 COPD	346.8 (271.9 to 412.5)	46.3 (39.4 to 53.3)	-3.6 (-8.3 to 0.7)
15 COPD	237 (185.9 to 281)	15 Oral disorders	321.2 (197.9 to 490.5)	-1.4 (-15.9 to 9.9)	-18.5 (-28.9 to -10.4)
16 Bipolar disorder	218 (129 to 330.1)	16 Alzheimer's disease	248.3 (176.8 to 331.6)	68.2 (54.9 to 82.6)	-13.1 (-19.2 to -6.8)
17 Stroke	208.3 (149.7 to 265.1)	17 Bipolar disorder	225.2 (133.5 to 342.9)	3.3 (-5.9 to 12.8)	0.6 (-6.7 to 8.8)
18 Psoriasis	205.4 (146.3 to 275.1)	18 Schizophrenia	211.9 (161.2 to 252.5)	13.8 (6.4 to 21.7)	0.2 (-6.4 to 7.5)
19 Asthma	201.6 (129.3 to 300.4)	19 Drug use disorders	205.1 (144.8 to 274)	4.5 (-7.6 to 18.8)	23 (8.3 to 39.5)
20 Drug use disorders	196.3 (135.2 to 261.8)	20 Psoriasis	202.1 (143.6 to 267.5)	-1.6 (-6.1 to 3.4)	-11.9 (-16.3 to -7.2)
21 Schizophrenia	186.3 (138.8 to 224)	21 Asthma	186.4 (120.1 to 275)	-7.5 (-17.4 to 6.9)	-5.2 (-16.5 to 11.7)
24 Alzheimer's disease	147.6 (106.2 to 194.4)	22 Stroke	186.1 (136.6 to 233.7)	-10.6 (-17.2 to -3.9)	-34.9 (-39.4 to -30.2)

Legend:
Communicable, maternal, neonatal and nutritional
Non-communicable
Injuries

Supp Figure 5. Changes in the rank of causes of disability-adjusted life years (DALYs; rate per 100,000) from 2010–2019

Leading causes 2010	DALYs per 100,000 2010
1 Ischemic heart disease	1609 (1469.7 to 1713.6)
2 Low back pain	1105.8 (788.5 to 1460.4)
3 Stroke	1091.8 (978.6 to 1175.8)
4 Lung cancer	1089.9 (1048 to 1121.5)
5 Diabetes	1046 (777.6 to 1353.5)
6 COPD	953.8 (845.1 to 1051)
7 Depressive disorders	929.1 (659.6 to 1260.4)
8 Headache disorders	820.3 (193.4 to 1737.3)
9 Alzheimer's disease	759.9 (337.6 to 1623.9)
10 Falls	700.3 (533 to 929.6)
11 Colorectal cancer	678 (632.2 to 711.6)
12 Gynecological diseases	610.8 (412.6 to 844.7)
13 Anxiety disorders	528.3 (377.3 to 712.6)
14 Other musculoskeletal	494.8 (342.6 to 682)
15 Endo/metab/blood/immune	475.1 (347.2 to 629.1)
16 Osteoarthritis	466.2 (226.6 to 957.8)
17 Age-related hearing loss	465.9 (313.3 to 669.5)
18 Cirrhosis	424.3 (402.6 to 446.2)
19 Road injuries	424.3 (381.2 to 473)
20 Chronic kidney disease	385.4 (340.7 to 422)
21 Breast cancer	385.1 (353.5 to 409.5)

Leading causes 2019	DALYs per 100,000 2019	Percent change rate of DALYs 2010-2019	Percent change age-standardized rate of DALYs 2010-2019
1 Ischemic heart disease	1613.6 (1474.9 to 1755.4)	0.3 (-3.7 to 4.5)	-16.6 (-19.9 to -13.2)
2 Diabetes	1145.5 (842.3 to 1513.6)	9.5 (3.2 to 16.7)	-2.5 (-8.3 to 4.3)
3 Lung cancer	1139.2 (1065.8 to 1202.2)	4.5 (-1.2 to 10.3)	-10 (-15.2 to -5.1)
4 Low back pain	1121 (806.2 to 1527.9)	1.4 (-6.5 to 11)	-3.5 (-10.9 to 5)
5 Stroke	1113.4 (989.8 to 1221.6)	2 (-3.6 to 8.1)	-15.3 (-20.1 to -10.1)
6 COPD	1103.2 (966.6 to 1233.9)	15.7 (5.8 to 27.2)	-4.1 (-12.2 to 5.1)
7 Depressive disorders	1021.5 (724.4 to 1392.7)	9.9 (3.7 to 16.5)	8.9 (2.6 to 14.4)
8 Alzheimer's disease	831.4 (378.2 to 1748.2)	9.4 (1.9 to 16.9)	-14.7 (-20 to -8.7)
9 Headache disorders	792.8 (189.2 to 1692.9)	-3.4 (-8.2 to 2.9)	-0.3 (-6 to 4.7)
10 Falls	777.8 (587.8 to 1040.4)	11.1 (8.9 to 13.2)	1.9 (-0.4 to 4.3)
11 Colorectal cancer	761 (695.8 to 820.6)	12.2 (5 to 19.5)	-4 (-10.4 to 2.4)
12 Gynecological diseases	585.2 (393.5 to 810)	-4.2 (-7.1 to -1.1)	0.4 (-1.5 to 2.3)
13 Osteoarthritis	541.1 (259.5 to 1106.3)	16.1 (13.4 to 18.7)	0.4 (-2 to 2.8)
14 Other musculoskeletal	538.2 (374 to 741.7)	8.8 (1.7 to 17.7)	4 (-2.3 to 11.3)
15 Age-related hearing loss	514.4 (346.3 to 735.5)	10.4 (5.2 to 15.6)	-3.3 (-7.3 to 0.6)
16 Endo/metab/blood/immune	507.7 (372.8 to 670.1)	6.8 (2 to 11.9)	-0.7 (-5 to 3.7)
17 Anxiety disorders	482.6 (325.8 to 682.1)	-8.7 (-20.5 to 3.1)	-7.4 (-19.5 to 6.1)
18 Chronic kidney disease	415.7 (365.8 to 463.3)	7.8 (0.6 to 16.4)	-12.9 (-18.2 to -6.8)
19 Breast cancer	405.8 (367.8 to 443.8)	5.4 (-2.4 to 13.5)	-7.7 (-14.5 to -0.1)
20 Cirrhosis	392.9 (362.5 to 424.1)	-7.4 (-13.8 to -0.6)	-19.6 (-25 to -13.5)
26 Road injuries	314.2 (279.7 to 351.5)	-26 (-29.3 to -22.9)	-24.8 (-28.7 to -21.2)

Legend:
Communicable, maternal, neonatal and nutritional
Non-communicable
Injuries

Supp Figure 6. Ranking in the causes of disability-adjusted life years (DALYs; rate per 100,000) in 2019 in: a) males; and b) females

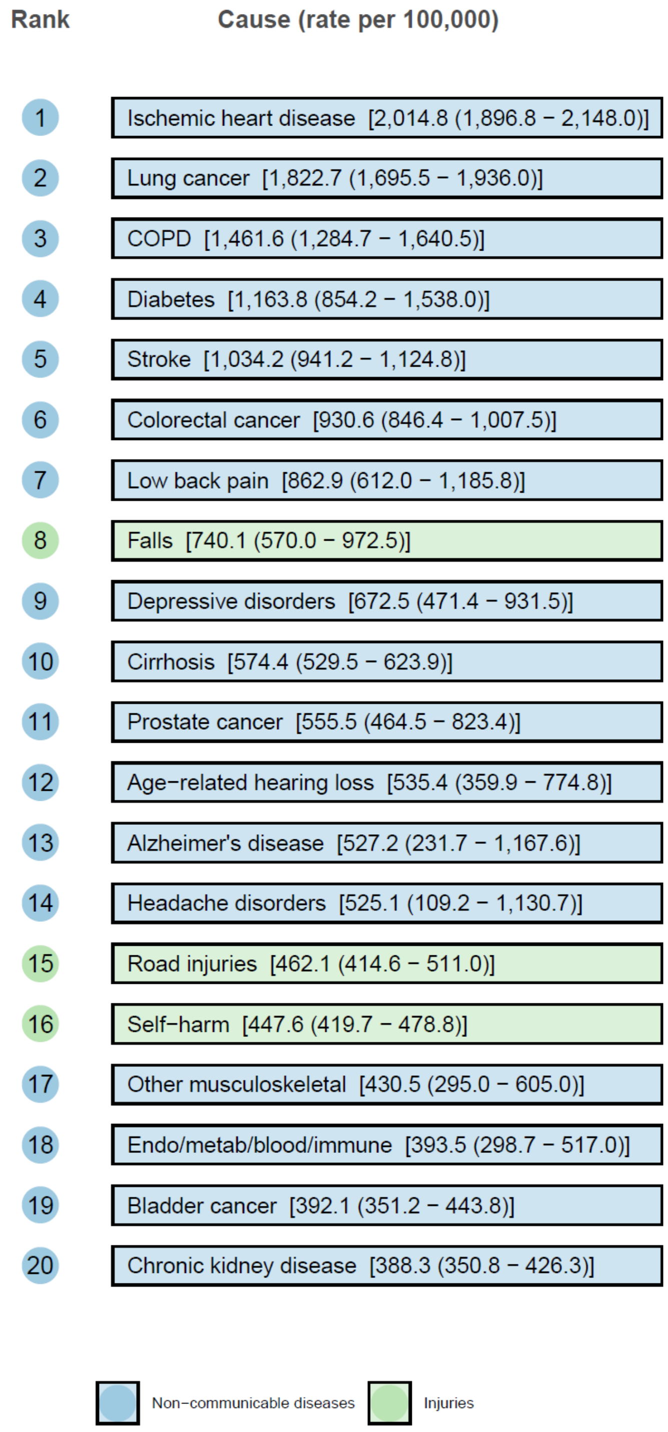

a)

Rank	Cause (rate per 100,000)
1	Low back pain [1,368.4 (980.3 − 1,864.2)]
2	Depressive disorders [1,356.0 (959.1 − 1,835.0)]
3	Ischemic heart disease [1,229.0 (1,051.3 − 1,400.3)]
4	Stroke [1,189.2 (1,027.5 − 1,331.7)]
5	Gynecological diseases [1,146.4 (770.9 − 1,586.7)]
6	Diabetes [1,128.0 (831.5 − 1,510.5)]
7	Alzheimer's disease [1,123.0 (517.2 − 2,324.9)]
8	Headache disorders [1,049.5 (268.0 − 2,212.6)]
9	Falls [814.0 (604.1 − 1,091.6)]
10	Breast cancer [785.6 (711.5 − 860.0)]
11	COPD [759.5 (593.5 − 905.1)]
12	Osteoarthritis [710.0 (336.2 − 1,482.6)]
13	Anxiety disorders [666.8 (435.2 − 940.6)]
14	Other musculoskeletal [641.4 (444.7 − 880.8)]
15	Endo/metab/blood/immune [617.1 (441.4 − 832.3)]
16	Colorectal cancer [598.4 (525.8 − 659.5)]
17	Age−related hearing loss [494.3 (333.0 − 704.8)]
18	Lung cancer [483.9 (432.0 − 532.9)]
19	Chronic kidney disease [441.9 (375.2 − 500.8)]
20	Neck pain [420.5 (275.5 − 609.9)]

Non−communicable diseases   Injuries

b)

Supp Figure 7. Ranking in the causes of disability-adjusted life years (DALYs; rate per 100,000) in all ages in 2019 in: a) Spain; b) high-income countries; and c) the world

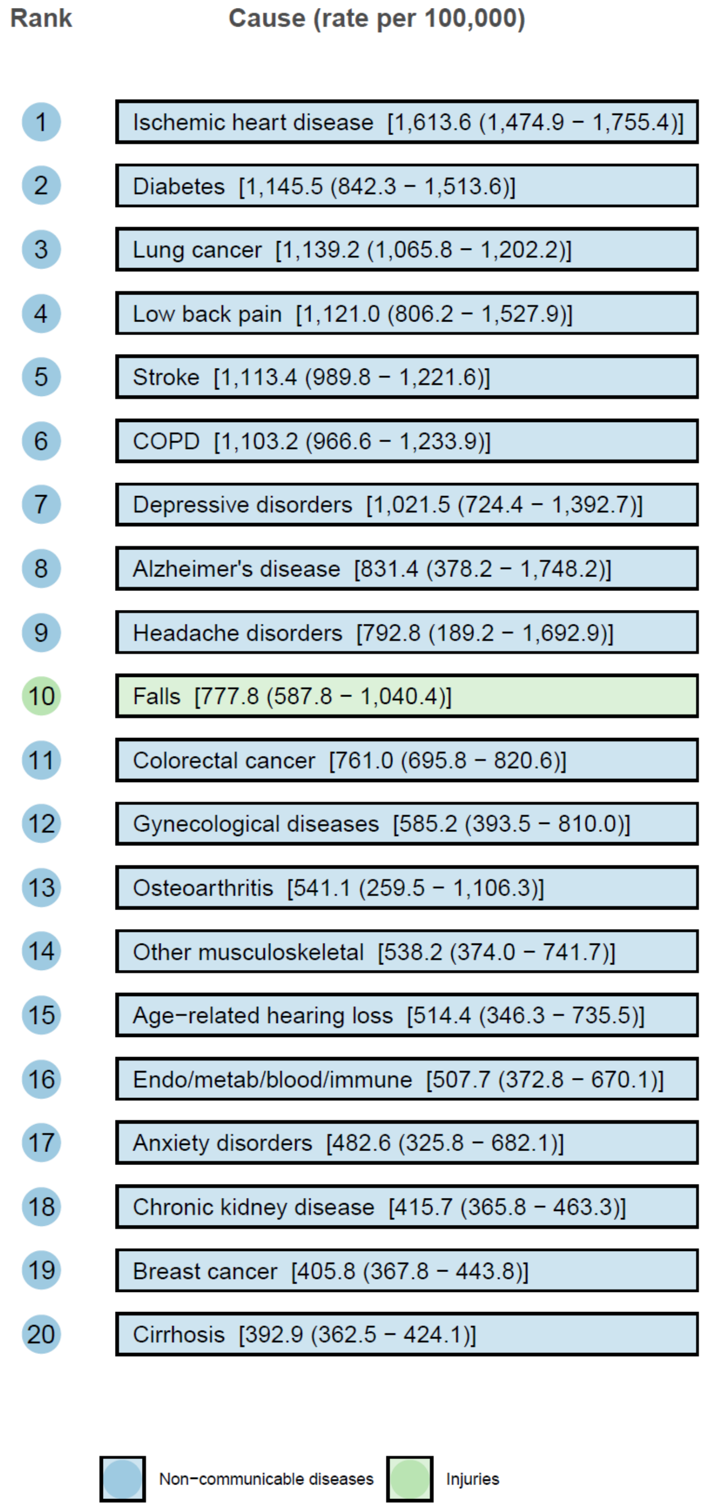

a)

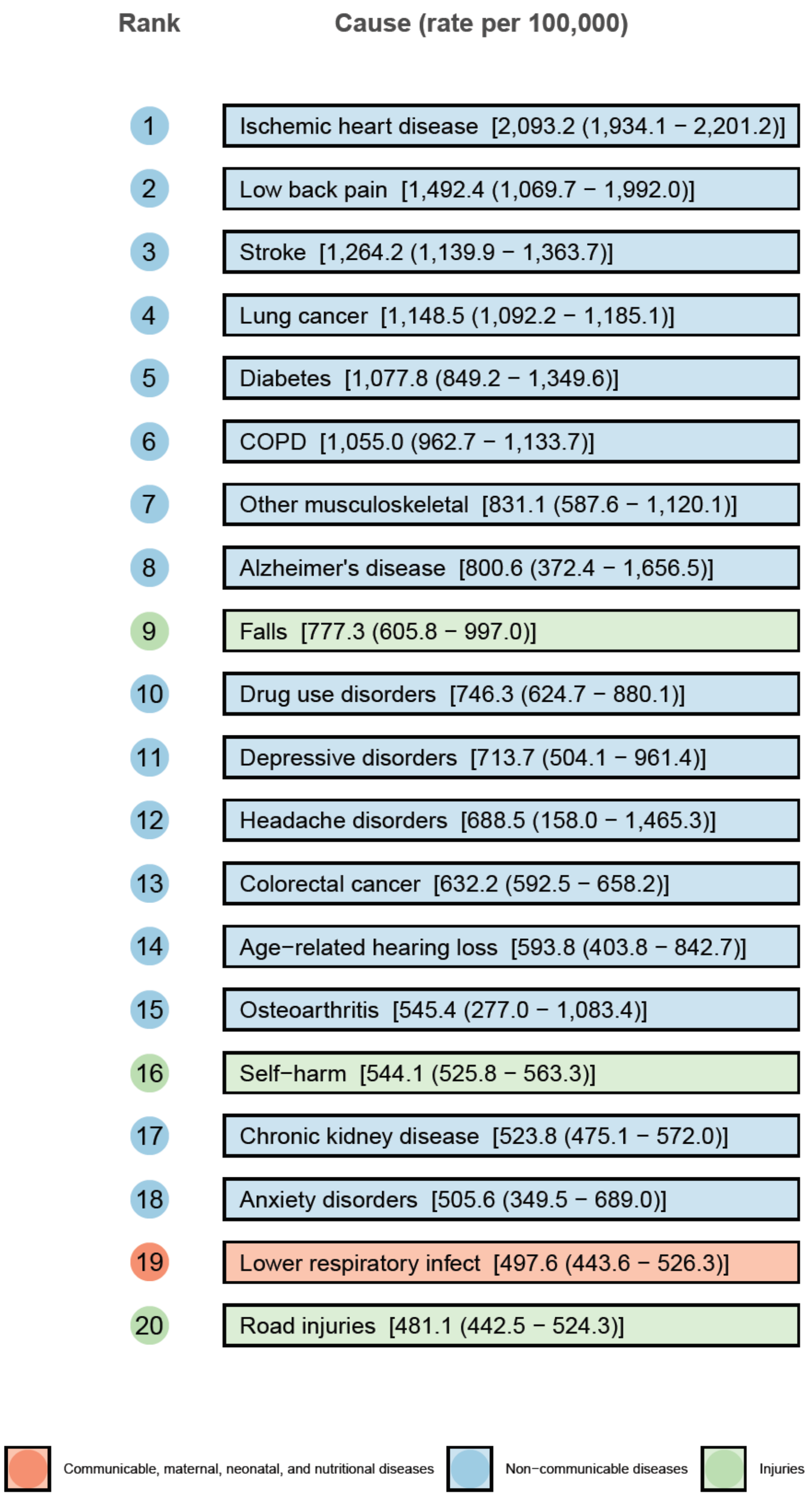
Rank
Cause (rate per 100,000)
1
Ischemic heart disease [2,093.2 (1,934.1 − 2,201.2)]
2
Low back pain [1,492.4 (1,069.7 − 1,992.0)]
3
Stroke [1,264.2 (1,139.9 − 1,363.7)]
4
Lung cancer [1,148.5 (1,092.2 − 1,185.1)]
5
Diabetes [1,077.8 (849.2 − 1,349.6)]
6
COPD [1,055.0 (962.7 − 1,133.7)]
7
Other musculoskeletal [831.1 (587.6 − 1,120.1)]
8
Alzheimer's disease [800.6 (372.4 − 1,656.5)]
9
Falls [777.3 (605.8 − 997.0)]
10
Drug use disorders [746.3 (624.7 − 880.1)]
11
Depressive disorders [713.7 (504.1 − 961.4)]
12
Headache disorders [688.5 (158.0 − 1,465.3)]
13
Colorectal cancer [632.2 (592.5 − 658.2)]
14
Age−related hearing loss [593.8 (403.8 − 842.7)]
15
Osteoarthritis [545.4 (277.0 − 1,083.4)]
16
Self−harm [544.1 (525.8 − 563.3)]
17
Chronic kidney disease [523.8 (475.1 − 572.0)]
18
Anxiety disorders [505.6 (349.5 − 689.0)]
19
Lower respiratory infect [497.6 (443.6 − 526.3)]
20
Road injuries [481.1 (442.5 − 524.3)]
Communicable, maternal, neonatal, and nutritional diseases
Non−communicable diseases
Injuries

b)

Rank	Cause (rate per 100,000)
1	Neonatal disorders [2,402.4 (2,074.3 − 2,824.3)]
2	Ischemic heart disease [2,352.6 (2,199.8 − 2,500.9)]
3	Stroke [1,851.2 (1,720.1 − 1,980.5)]
4	Lower respiratory infect [1,256.1 (1,096.9 − 1,461.5)]
5	Diarrheal diseases [1,045.8 (844.9 − 1,331.4)]
6	COPD [962.0 (881.5 − 1,036.4)]
7	Road injuries [942.2 (837.9 − 1,036.4)]
8	Diabetes [916.1 (771.7 − 1,087.9)]
9	Low back pain [823.1 (581.6 − 1,101.0)]
10	Congenital defects [682.2 (563.5 − 846.8)]
11	HIV/AIDS [615.6 (551.0 − 719.2)]
12	Tuberculosis [607.8 (553.3 − 664.0)]
13	Depressive disorders [605.7 (425.6 − 824.5)]
14	Headache disorders [602.5 (126.3 − 1,294.5)]
15	Malaria [600.2 (303.8 − 1,035.4)]
16	Cirrhosis [597.0 (556.1 − 640.4)]
17	Lung cancer [592.7 (546.7 − 637.7)]
18	Chronic kidney disease [536.9 (494.9 − 582.1)]
19	Other musculoskeletal [522.4 (364.7 − 717.0)]
20	Age−related hearing loss [520.0 (354.0 − 738.4)]

Communicable, maternal, neonatal, and nutritional diseases | Non−communicable diseases | Injuries

c)

Supp Figure 8. Changes in the rank of the causes of years of life lost (YLLs) (rate per 100,000) from 1990 to 2019

Leading causes 1990	YLLs per 100,000 1990	Leading causes 2019	YLLs per 100,000 2019	Percent change rate of YLLs 1990-2019	Percent change age-standardized rate of YLLs 1990-2019
1 Ischemic heart disease	2450 (2311.3 to 2532.1)	1 Ischemic heart disease	1525.8 (1387.8 to 1662.6)	-37.7 (-41.4 to -32.8)	-60.4 (-62.3 to -57.7)
2 Stroke	1811.7 (1695.1 to 1895.6)	2 Lung cancer	1122.9 (1053.2 to 1185)	10.7 (3.5 to 17.3)	-17.6 (-22.8 to -12.6)
3 Road injuries	1193.8 (1159.8 to 1222.8)	3 Stroke	927.3 (807.5 to 1027.9)	-48.8 (-53.2 to -44.1)	-69.8 (-72 to -67.2)
4 Lung cancer	1014.8 (987 to 1045.5)	4 COPD	756.4 (640.8 to 859.4)	4.3 (-9.7 to 19)	-38.8 (-45.5 to -30.8)
5 Cirrhosis	764.2 (737.1 to 790.4)	5 Colorectal cancer	707.4 (648.6 to 756.1)	35.1 (26.2 to 44.6)	-6.5 (-12.3 to -0.3)
6 COPD	725.1 (672.5 to 769.4)	6 Alzheimer's disease	583 (147.8 to 1480.2)	71.3 (54.9 to 93.9)	-17.6 (-23 to -10.3)
7 Colorectal cancer	523.5 (501.4 to 541.4)	7 Cirrhosis	385.2 (354.8 to 416.2)	-49.6 (-53.5 to -45.2)	-63.5 (-66.3 to -60.5)
8 Stomach cancer	446.7 (427.3 to 462.5)	8 Breast cancer	355.9 (326.5 to 380.8)	-17.7 (-23.7 to -12.1)	-41.6 (-45.6 to -37.7)
9 Breast cancer	432.7 (415.5 to 447.5)	9 Lower respiratory infect	338.8 (293.8 to 376.2)	-11.9 (-20.8 to -3.4)	-53.5 (-57.7 to -49.4)
10 Diabetes	408.5 (382 to 427.6)	10 Chronic kidney disease	330.2 (285.4 to 369.6)	6.5 (-5.1 to 18.4)	-41.8 (-46.9 to -36)
11 Lower respiratory infect	384.4 (359.4 to 403.7)	11 Pancreatic cancer	320.4 (291.6 to 349.9)	54.1 (40.6 to 68.4)	12.4 (3 to 23)
12 Self-harm	349.1 (336.4 to 362.2)	12 Self-harm	281.4 (265.6 to 298.4)	-19.4 (-25.4 to -13.1)	-24.3 (-29.7 to -18.3)
13 Alzheimer's disease	340.3 (82.6 to 908.9)	13 Stomach cancer	272 (251 to 293.6)	-39.1 (-43.3 to -34.7)	-56.7 (-59.7 to -53.5)
14 Neonatal disorders	332.1 (300.1 to 377.2)	14 Diabetes	260.4 (227.9 to 283.3)	-36.3 (-41.6 to -30.8)	-60.8 (-63.7 to -57.9)
15 Congenital defects	320.3 (230.1 to 350.7)	15 Prostate cancer	234.5 (197.8 to 347.3)	9.2 (-6.5 to 45.3)	-29.8 (-40.1 to -5.1)
16 Chronic kidney disease	309.9 (287.7 to 329)	16 Liver cancer	209.3 (189 to 230)	69.4 (52.8 to 86.8)	26.3 (13.5 to 39.9)
17 HIV/AIDS	304.1 (295.7 to 313)	17 Road injuries	209.1 (195.9 to 221.5)	-82.5 (-83.7 to -81.4)	-81.4 (-82.7 to -80.2)
18 Prostate cancer	214.8 (160.6 to 258.2)	18 Bladder cancer	207.3 (186.8 to 230.4)	8.4 (-1.5 to 20.7)	-26.9 (-33.3 to -18.3)
19 Cardiomyopathy	213 (165.6 to 232)	19 Cardiomyopathy	192.5 (153.6 to 219.2)	-9.6 (-18.9 to 1.4)	-42.9 (-49.1 to -35.7)
20 Leukemia	208.8 (199.9 to 217)	20 Hypertensive heart disease	178.3 (101.3 to 209.2)	132.6 (19.5 to 175)	20.2 (-36 to 40.6)
21 Pancreatic cancer	207.9 (198.9 to 216)	22 Leukemia	170.9 (156.6 to 183.5)	-18.2 (-24.6 to -11.5)	-39.4 (-44.2 to -34.3)
22 Bladder cancer	191.3 (181.4 to 199.8)	36 Neonatal disorders	93.2 (74.7 to 114)	-71.9 (-79 to -64.6)	-64.2 (-73.3 to -54.8)
31 Liver cancer	123.6 (118 to 128.8)	37 Congenital defects	92.1 (79.3 to 118.3)	-71.3 (-76.4 to -50.7)	-67.8 (-74.5 to -41.5)
40 Hypertensive heart disease	76.7 (67.2 to 96.6)	48 HIV/AIDS	61.4 (57.9 to 64.9)	-79.8 (-81 to -78.4)	-84.2 (-85.1 to -83.1)

Legend:
Communicable, maternal, neonatal and nutritional
Non-communicable
Injuries

Supp Figure 9. Ranking in the causes of years of life lost (YLLs; rate per 100,000) in 2019 in: a) males; and b) female

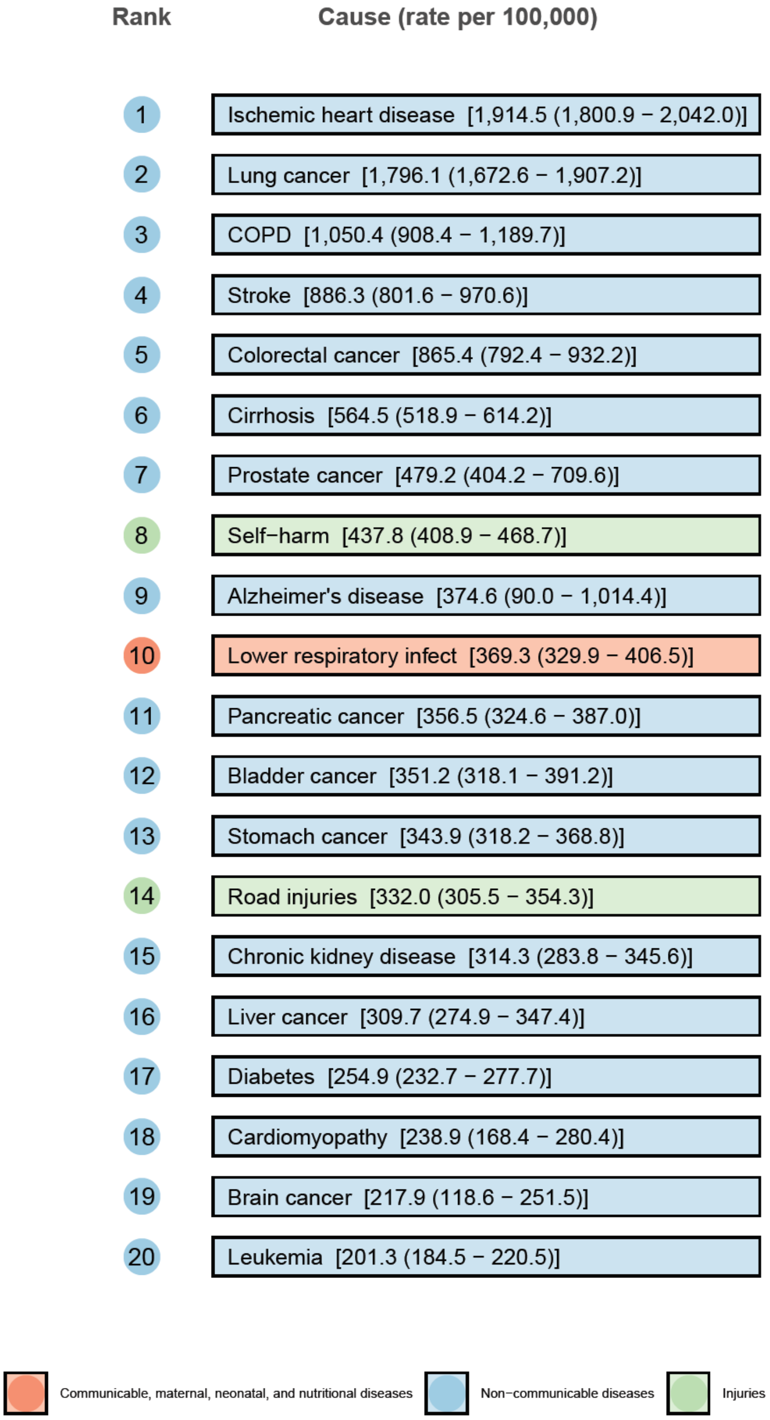

a)

b)

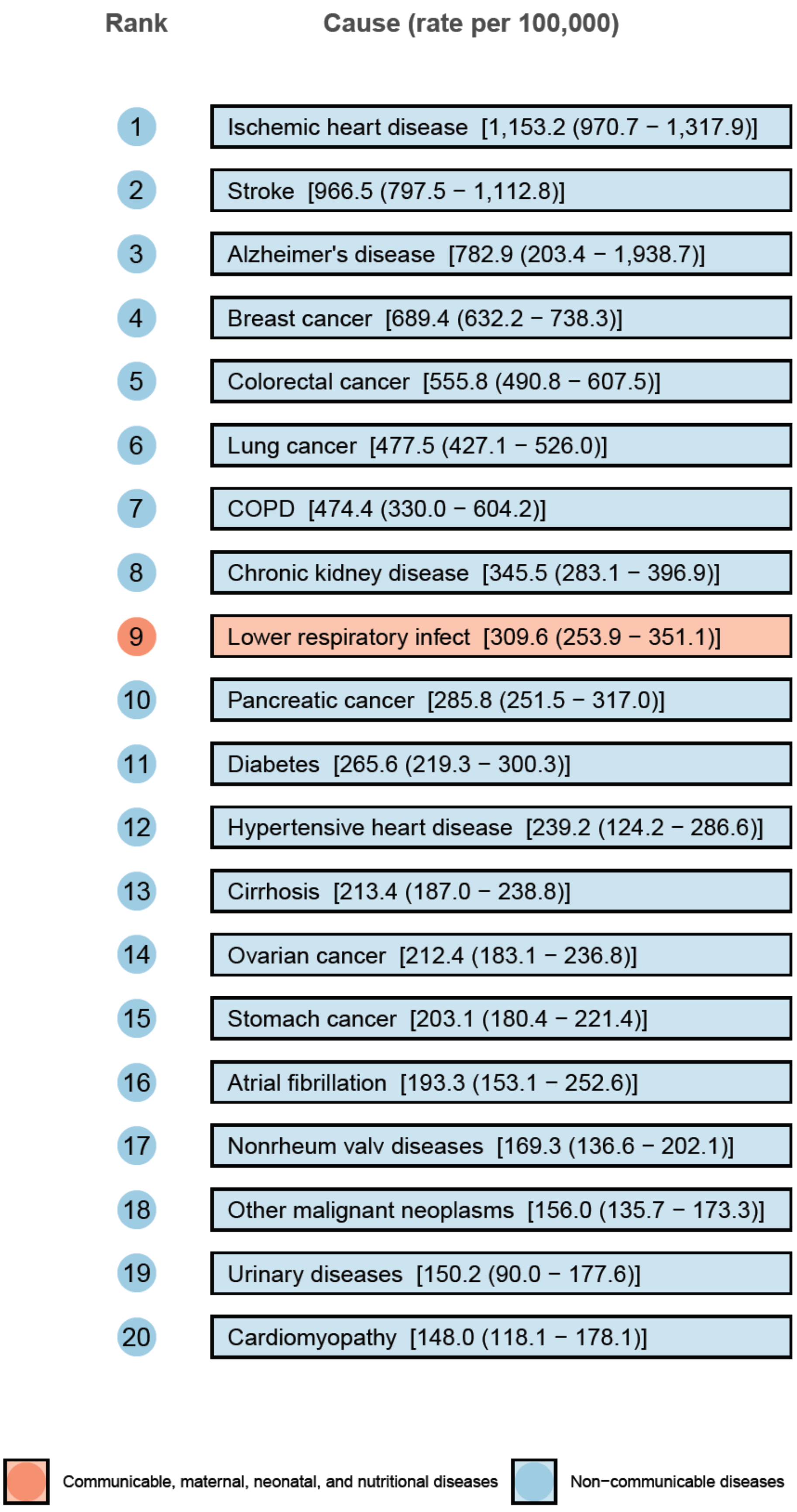
Rank
Cause (rate per 100,000)
1
Ischemic heart disease [1,153.2 (970.7 − 1,317.9)]
2
Stroke [966.5 (797.5 − 1,112.8)]
3
Alzheimer's disease [782.9 (203.4 − 1,938.7)]
4
Breast cancer [689.4 (632.2 − 738.3)]
5
Colorectal cancer [555.8 (490.8 − 607.5)]
6
Lung cancer [477.5 (427.1 − 526.0)]
7
COPD [474.4 (330.0 − 604.2)]
8
Chronic kidney disease [345.5 (283.1 − 396.9)]
9
Lower respiratory infect [309.6 (253.9 − 351.1)]
10
Pancreatic cancer [285.8 (251.5 − 317.0)]
11
Diabetes [265.6 (219.3 − 300.3)]
12
Hypertensive heart disease [239.2 (124.2 − 286.6)]
13
Cirrhosis [213.4 (187.0 − 238.8)]
14
Ovarian cancer [212.4 (183.1 − 236.8)]
15
Stomach cancer [203.1 (180.4 − 221.4)]
16
Atrial fibrillation [193.3 (153.1 − 252.6)]
17
Nonrheum valv diseases [169.3 (136.6 − 202.1)]
18
Other malignant neoplasms [156.0 (135.7 − 173.3)]
19
Urinary diseases [150.2 (90.0 − 177.6)]
20
Cardiomyopathy [148.0 (118.1 − 178.1)]
Communicable, maternal, neonatal, and nutritional diseases
Non−communicable diseases

Supp Figure 10. Changes in the rank of risk factors for years lived with disability (YLDs; rate per 100,000) from 2010–2019

Leading risks 2010	YLDs per 100,000 2010	Leading risks 2019	YLDs per 100,000 2019	Percent change rate of YLDs 2010-2019	Percent change age-standardized rate of YLDs 2010-2019
1 High FPG (continuous)	880.3 (592.9 to 1209.1)	1 High FPG (continuous)	987.4 (660.5 to 1372.4)	12.2 (4.6 to 21.2)	0.9 (-6.1 to 9.1)
2 High BMI (adult)	874.1 (513.5 to 1332.5)	2 High BMI (adult)	955.8 (565.2 to 1470)	9.4 (3.3 to 16.4)	-1.8 (-7.2 to 4.5)
3 Smoking	777.4 (568.4 to 998.3)	3 Smoking	838.8 (618.4 to 1080.9)	7.9 (1.3 to 15.3)	-3 (-9.2 to 3.6)
4 Drug use	189.1 (132.8 to 250.3)	4 Drug use	207.7 (146.9 to 277.4)	9.8 (-4.4 to 27.1)	20.9 (4.1 to 42)
5 Occupational ergonomic	166.6 (116.3 to 225.9)	5 Occupational ergonomic	160.7 (109.7 to 224.1)	-3.6 (-14.3 to 8.8)	-4.8 (-15.6 to 6.1)
6 Particulate matter	124.1 (74.3 to 188.6)	6 High processed meat	131 (76.9 to 198.8)	11.7 (3.2 to 22.3)	-0.4 (-8.3 to 8.8)
7 High processed meat	117.3 (68.3 to 175.8)	7 High red meat	125.7 (80 to 183.2)	10.5 (1.6 to 19.8)	-1 (-8.8 to 7.4)
8 High red meat	113.7 (72.9 to 166.7)	8 Particulate matter	111.1 (63.5 to 174.9)	-10.5 (-20.9 to -2.1)	-20.6 (-30 to -13.1)
9 Occupational injury	93.9 (61 to 135)	9 Low birth weight & short gestation	85.2 (64.9 to 109.6)	-6.2 (-21.7 to 14)	-3.8 (-20.3 to 16.5)
10 Low birth weight & short gestation	90.8 (68.4 to 114.2)	10 Second-hand smoke	84 (43.9 to 134.9)	7.4 (-1.5 to 18.5)	-4.4 (-12.3 to 5.2)
11 Second-hand smoke	78.2 (39.8 to 124.2)	11 Occupational injury	79.1 (46.7 to 124.6)	-15.7 (-37.8 to 10.7)	-14.6 (-36.5 to 10.8)
12 Low whole grains	64 (33.2 to 99.8)	12 Low whole grains	71.2 (37.2 to 110.8)	11.3 (4.9 to 18)	-1.5 (-7.3 to 4.6)
13 High FPG (categorical)	59 (19 to 126.4)	13 High FPG (categorical)	66.1 (21 to 141.5)	12 (1.5 to 22.2)	-6.8 (-15.6 to 2.3)
14 Iron deficiency	50.6 (27.8 to 85.5)	14 High sweetened beverages	48.5 (20.8 to 80.2)	25.3 (7.4 to 64.3)	14 (-2.8 to 52.7)
15 Lead	46.4 (25.1 to 72.3)	15 Child growth failure	48 (30.3 to 70.5)	8.5 (-6.6 to 28.3)	2.8 (-12.1 to 21.9)
16 Child growth failure	44.2 (28.1 to 65.2)	16 Iron deficiency	46.7 (25.6 to 80.3)	-7.6 (-25.8 to 14.4)	-7.3 (-29.1 to 20.5)
17 High sweetened beverages	38.7 (15.7 to 65)	17 Lead	41.2 (21.9 to 65.6)	-11.2 (-17 to -5.7)	-19.2 (-24.8 to -13)
18 Low fibre	36.4 (18.1 to 58.7)	18 Low fibre	39.3 (19.7 to 64.3)	7.9 (-1.8 to 17.4)	-3.4 (-12.2 to 5.5)
19 Bullying	35.3 (7.9 to 85.9)	19 Childhood sexual abuse	36.2 (17.3 to 63.8)	18.4 (2.7 to 36.2)	20.4 (5.9 to 38.8)
20 Occupational noise	33 (22.2 to 48)	20 Occupational noise	34.7 (23.3 to 50.1)	5.1 (-0.4 to 11.1)	-5.4 (-10.2 to -0.5)
21 Childhood sexual abuse	30.6 (15 to 55.7)	21 Bullying	33 (7.9 to 77.6)	-6.6 (-17.5 to 10.5)	3.2 (-7.3 to 14.4)

Legend:
Environmental/occupational risks
Behavioral risks
Metabolic risks

Supp Figure 11. Changes in the rank of risk factors for years lived with disability (YLDs; rate per 100,000) in 2019 in: a) males; and b) females

a)

Leading risks 2010	YLDs per 100,000 2010	Leading risks 2019	YLDs per 100,000 2019	Percent change rate of YLDs 2010-2019	Percent change age-standardized rate of YLDs 2010-2019
1 Smoking	1002.4 (725.9 to 1281.2)	1 Smoking	1021.8 (768.5 to 1300.5)	1.9 (-5 to 10.6)	-11 (-17.3 to -3.4)
2 High FPG (continuous)	856.2 (573.5 to 1193)	2 High FPG (continuous)	1006.9 (671.8 to 1401.9)	17.6 (7.4 to 29.5)	3.2 (-5.9 to 13.7)
3 High BMI (adult)	767.3 (441.2 to 1194.7)	3 High BMI (adult)	850.2 (474.3 to 1344.3)	10.8 (1.9 to 21.2)	-2.1 (-10.1 to 6.7)
4 Drug use	245 (170.8 to 326.9)	4 Drug use	252.3 (175.4 to 341.5)	3 (-11.2 to 20.1)	13.8 (-3.1 to 34)
5 Occupational ergonomic	178.5 (123.7 to 241.3)	5 Occupational ergonomic	154.8 (106.8 to 221.1)	-13.3 (-25.3 to 0.6)	-14.1 (-26 to -1.4)
6 Occupational injury	167.6 (107.3 to 243.2)	6 Occupational injury	136.8 (79.9 to 218.5)	-18.4 (-40 to 8.7)	-17.5 (-39.2 to 8.5)
7 Particulate matter	124.3 (73.6 to 190.6)	7 High processed meat	134.5 (79.1 to 207.4)	17.2 (5.5 to 30.2)	2.1 (-8.2 to 13.9)
8 High processed meat	114.8 (67.3 to 170.3)	8 High red meat	125.7 (78.7 to 187.9)	14.8 (2 to 28.1)	0.7 (-10.2 to 12.2)
9 High red meat	109.4 (69.5 to 163.2)	9 Particulate matter	115.8 (64.7 to 184)	-6.8 (-18.5 to 3.1)	-19.3 (-29.4 to -10.7)
10 Low birth weight & short gestation	91.6 (68.8 to 116.5)	10 Second-hand smoke	90.7 (48.1 to 147.6)	11.2 (-1.8 to 26.5)	-3.3 (-14.8 to 9.4)
11 Second-hand smoke	81.6 (41.7 to 130.7)	11 Low birth weight & short gestation	85.4 (64.6 to 110.1)	-6.8 (-23.7 to 13.7)	-3.9 (-21.6 to 17.1)
12 Low whole grains	65.8 (34.1 to 101.2)	12 Low whole grains	75.8 (39 to 119.3)	15.3 (7.3 to 24.2)	-0.4 (-7.3 to 7.6)
13 Occupational noise	49.2 (33.1 to 71.7)	13 High FPG (categorical)	52.6 (16 to 113.1)	18.4 (4.1 to 31.4)	-3.5 (-14.9 to 7.3)
14 Lead	46.9 (25.7 to 74)	14 Occupational noise	51 (34 to 73.4)	3.7 (-2.9 to 10.8)	-8.3 (-13.8 to -2.5)
15 Child growth failure	45.3 (26.6 to 68.9)	15 Child growth failure	49.3 (29.9 to 74)	9 (-14 to 37.3)	3.5 (-18.9 to 31)
16 High FPG (categorical)	44.4 (13.6 to 93.4)	16 Occupational particulates	48.5 (33 to 66.6)	19.5 (-4.1 to 59.2)	0.3 (-19.5 to 32.6)
17 Occupational particulates	40.6 (26.6 to 57.5)	17 High sweetened beverages	47.5 (19.7 to 78.6)	30.3 (7.3 to 70.2)	15.8 (-5.7 to 52.8)
18 Iron deficiency	39.5 (18.6 to 76.1)	18 Lead	41.2 (22.2 to 65.3)	-12.2 (-20.2 to -4.9)	-20.1 (-27.7 to -11.7)
19 High sweetened beverages	36.5 (15.4 to 60.9)	19 Low fibre	39.6 (19 to 65.9)	11.1 (-3.7 to 25.1)	-2.6 (-14.8 to 9.7)
20 Low fibre	35.6 (17.9 to 58.7)	20 Iron deficiency	36.8 (16.7 to 74.9)	-6.8 (-36.4 to 36.9)	-5.5 (-38.5 to 45.4)

Legend:
Environmental/occupational risks
Behavioral risks
Metabolic risks

b)

Leading risks 2010	YLDs per 100,000 2010	Leading risks 2019	YLDs per 100,000 2019	Percent change rate of YLDs 2010-2019	Percent change age-standardized rate of YLDs 2010-2019
1 High BMI (adult)	978.3 (589.9 to 1479.1)	1 High BMI (adult)	1057.1 (634.1 to 1576.9)	8.1 (1 to 15.5)	-1.2 (-7.5 to 5.8)
2 High FPG (continuous)	903.9 (607.2 to 1244)	2 High FPG (continuous)	968.8 (643.8 to 1350.2)	7.2 (-2.2 to 17.5)	-1.3 (-10.3 to 8.2)
3 Smoking	557.7 (401.7 to 722)	3 Smoking	663.3 (476.2 to 875.8)	18.9 (9.8 to 28.7)	8.9 (0.7 to 17.9)
4 Occupational ergonomic	155 (108.6 to 207.5)	4 Occupational ergonomic	166.3 (112.8 to 230.9)	7.3 (-5.4 to 22.2)	5.4 (-7 to 19.2)
5 Drug use	134.5 (94 to 179.5)	5 Drug use	164.9 (114.7 to 225.7)	22.6 (0.9 to 48.7)	34.6 (9.4 to 65.6)
6 Particulate matter	124 (73.7 to 188)	6 High processed meat	127.6 (75.3 to 196.5)	6.5 (-4 to 18.2)	-2.9 (-12.7 to 7.8)
7 High processed meat	119.8 (69.9 to 179.9)	7 High red meat	125.7 (81.2 to 182.6)	6.6 (-5.2 to 17.9)	-2.5 (-12.6 to 7.2)
8 High red meat	117.9 (76.5 to 171.2)	8 Particulate matter	106.7 (60.5 to 166.8)	-14 (-24.7 to -4.9)	-22.1 (-31.7 to -13.9)
9 Low birth weight & short gestation	90 (67.3 to 115.8)	9 Low birth weight & short gestation	85 (65 to 110.2)	-5.6 (-21.6 to 15.5)	-3.5 (-20.2 to 19.3)
10 Second-hand smoke	74.8 (38.8 to 117.5)	10 High FPG (categorical)	79 (23.1 to 173.5)	7.9 (-3.1 to 19.9)	-8.4 (-18.1 to 2)
11 High FPG (categorical)	73.2 (21.6 to 156.3)	11 Second-hand smoke	77.5 (40.5 to 122)	3.5 (-6.9 to 14.8)	-5.5 (-14.6 to 4.4)
12 Low whole grains	62.3 (31.6 to 98.2)	12 Low whole grains	66.8 (34.5 to 104.7)	7.3 (0 to 15)	-3 (-9.7 to 4.4)
13 Iron deficiency	61.3 (32.2 to 108.1)	13 Iron deficiency	56.2 (30.4 to 99.3)	-8.4 (-28.9 to 18.9)	-9.4 (-30.1 to 18.7)
14 Lead	45.8 (25 to 71.6)	14 IPV (exposure)	50.7 (0.2 to 124)	20 (3.5 to 38.7)	23.7 (5.6 to 44.6)
15 Bullying	45.8 (10.1 to 110.9)	15 High sweetened beverages	49.4 (21.6 to 84.5)	20.9 (-3.3 to 67.6)	12.7 (-9.7 to 57.2)
16 Child growth failure	43.2 (27.2 to 64.9)	16 Child growth failure	46.7 (28.8 to 69.5)	8 (-12.2 to 33.5)	2.1 (-17.2 to 26.2)
17 IPV (exposure)	42.2 (0.1 to 101.8)	17 Bullying	42.1 (10 to 97.7)	-8 (-20.7 to 12)	2.7 (-10.5 to 16.6)
18 High sweetened beverages	40.9 (16.5 to 69.8)	18 Lead	41.2 (21.6 to 64.7)	-10.2 (-17.5 to -3.4)	-18.5 (-25.9 to -10.5)
19 Low fibre	37.2 (18.2 to 59.7)	19 Low fibre	39 (19.1 to 64.2)	4.9 (-6.5 to 18.2)	-4.2 (-14.7 to 7.5)
20 Childhood sexual abuse	30.9 (14.9 to 54.7)	20 Childhood sexual abuse	36 (16.5 to 64.6)	16.3 (-1.3 to 36.8)	19.8 (1.5 to 42.1)

Legend:
Environmental/occupational risks
Behavioral risks
Metabolic risks
